# Great genetic diversity of vector-borne bacteria and protozoan in wild rodents from Guangxi, China

**DOI:** 10.1371/journal.pntd.0012159

**Published:** 2024-05-13

**Authors:** Jing Xue, Si-Si Chen, Rui Jian, Guo-Qing Chen, Xincheng Qin, Miao Lu, Wen Wang, Guang-Cheng Xie, Luanying Du, Kun Li, Wen-Ping Guo

**Affiliations:** 1 College of Basic Medicine, Chengde Medical University, Chengde, Hebei, China; 2 Yancheng Center for Disease Control and Prevention, Yancheng, Jiangsu, China; 3 National Institute for Communicable Disease Control and Prevention, Beijing, China; Insitut Pasteur de Tunis, TUNISIA

## Abstract

**Background:**

Rodents are recognized as the hosts of many vector-borne bacteria and protozoan parasites and play an important role in their transmission and maintenance. Intensive studies have focused on their infections in vectors, especially in ticks, however, vector-borne bacterial and protozoan infections in rodents are poorly understood although human cases presenting with fever may due to their infection have been found.

**Methods:**

From May to October 2019, 192 wild rodents were trapped in wild environment of Guangxi Province, and the spleen samples were collected to reveal the presence of vector-borne bacterial and protozoan infections in them. The microorganisms in rodents were identified by detecting their DNA using (semi-)nested PCR. All the PCR products of the expected size were subjected to sequencing, and then analyzed by BLASTn. Furthermore, all the recovered sequences were subjected to nucleotide identity and phylogenetic analyses.

**Results:**

As a result, 192 rodents representing seven species were captured, and *Bandicota indica* were the dominant species, followed by *Rattus andamanensis*. Based on the (semi-)nested PCR, our results suggested that *Anaplasma bovis*, *Anaplasma capra*, *Anaplasma ovis*, *Anaplasma phagocytophilum*, “*Candidatus* Neoehrlichia mikurensis”, “*Candidatus* E. hainanensis”, “*Candidatus* E. zunyiensis”, three uncultured *Ehrlichia* spp., *Bartonella coopersplainsensis*, *Bartonella tribocorum*, *Bartonella rattimassiliensis*, *Bartonella silvatica*, two uncultured *Bartonella* spp., *Babesia microti* and diverse *Hepatozoon* were identified in six rodent species. More importantly, six species (including two *Anaplasma*, two *Bartonella*, “*Ca*. N. mikurensis” and *Bab*. *microti*) are zoonotic pathogens except *Anaplasma bovis* and *Anaplasma ovis* with zoonotic potential. Furthermore, dual infection was observed between different microorganisms, and the most common type of co-infection is between “*Ca*. N. mikurensis” and other microorganisms. Additionally, potential novel *Bartonella* species and *Hepatozoon* species demonstrated the presence of more diverse rodent-associated *Bartonella* and *Hepatozoon*.

**Conclusions:**

The results in this work indicated great genetic diversity of vector-borne infections in wild rodents, and highlighted the potential risk of human pathogens transmitted from rodents to humans through vectors.

## Author summary

Rodents are the important hosts of many pathogens, especially vector-borne pathogens, and can harbor and transmit them to humans and cause diseases. In China, little information is known about the infection of vector-borne pathogens in rodents although the rodent species are very diverse. In this study, seven wild rodent species were collected to identify the genetic diversity of vector-borne bacteria and protozoan. Interestingly, diverse vector-borne microorganisms, including four *Anaplasma*, five *Ehrlichia*, “*Candidatus* Neoehrlichia mikurensis”, six *Bartonella*, *Babesia microti* and diverse *Hepatozoon* were identified in six rodent species. Furthermore, two potential novel *Bartonella* species were detected. All these data suggested great genetic diversity of vector-borne pathogens infections in rodents. Of them, six species (including two *Anaplasma*, two *Bartonella*, “*Ca*. N. mikurensis” and *Bab*. *microti*) are pathogenic to humans except *A*. *bovis* and *A*. *ovis* with zoonotic potential, suggesting significant risks to local human population.

## Introduction

As the most abundant and diverse mammals, rodents are notorious due to their ability to host and transmit a wide range of zoonotic pathogens, such as bacteria, viruses, and parasites [1]. Among them, infection with some pathogens can cause serious diseases in humans, including *Yersina pestis* causing plague [2], orthohantaviruses causing hemorrhagic fever with renal syndrome (HFRS) and hantavirus cardiopulmonary syndrome (HCPS) [3], Lassa virus causing Lassa fever (LF) [4], *Francisella tularensis* causing tularemia [5], etc. Except for the direct route, rodent-associated causative agents can be transmitted indirectly to humans and animals mainly through blood-feeding ectoparasitic arthropods [6]. As the important hosts infested by immature ticks, rodents are considered to be the competent reservoirs of many vector-borne pathogens. They play an important role in maintaining and transmitting a great extensive vector-borne zoonotic and veterinary bacterial and protozoan pathogens, such as Anaplasmataceae, *Bartonella*, *Babesia* and *Hepatozoon* [1].

Many tick-borne Anaplasmataceae bacteria are pathogenic to humans and animals [7]. Specifically, pathogens that cause diseases mainly include some members within *Anaplasma*, *Ehrlichia* and *Candidatus* Neoehrlichia [8]. Because no transovarial transmission of *Anaplasma*, *Candidatus* Neoehrlichia, and *Ehrlichia* was observed in ticks [9], vertebrates including rodents are necessary to be the hosts of them. Many species within these genera, including *A*. *phagocytophilum* and *Ehrlichia chaffeensis* causing human monocytic ehrlichiosis (HME) and human granulocytic ehrlichiosis (HGE), respectively, have been identified in rodents [1]. Contrary to the family Anaplasmataceae, *Bartonella* spp. are facultative intracellular alphaproteobacteria and comprise a large number of vector-borne bacteria [10]. To date, more than 40 species including eight zoonotic pathogens have been identified in a wide range of rodents [11]. Therefore, rodents are recognized as the main natural reservoirs of the genus *Bartonella* [11]. In China, 22 rodent-associated *Bartonella* species, including eight pathogenic to humans, have been found so far, indicating a potential risk to humans [12].

Genera *Babesia* and *Hepatozoon* belong to Apicomplexa of Protozoa. Some species within the genus *Babesia* are uncommon zoonotic agents and are transmitted to humans through blood feeding by ticks [13,14]. Rodents are considered to be the main reservoirs of *Babesia*, especially for *Bab*. *microti*, which is commonly distributed throughout the world and identified in diverse rodent species [13]. Genus *Hepatozoon* is composed of more than three hundred species, and invertebrate animals and vertebrate animals are used as its definitive and intermediate hosts, respectively [15,16]. Its transmission from vectors to vertebrates occurs when the intermediate host ingests the definitive host, differing from most other vector-borne protozoal and bacterial pathogens [17]. Definitely, rodents have been confirmed to be the intermediate hosts of *H*. *ayorgbor* [18]. In addition, *Hepatozoon* spp. have been identified in diverse rodents although their role as intermediate or paratenic hosts to *Hepatozoon* spp. is uncertain [19,20].

Guangxi Zhuang Autonomous Region is located in South China, and its topography is characterized by mountains and hills. The subtropical climate and dense vegetation are conducive to the survival of various wild rodents. As the hosts of many vector-borne microorganisms including human pathogens, molecular surveys on vector-borne bacterial and protozoan infections in rodents, including Anaplasmataceae, *Bartonella* and *Babesia*, have been performed in many parts of China in the past 20 years [12,19,21–26]. In recent years, an increasing number of patients presenting with fever but lacking a confirmed viral etiology has been observed mainly in Guangxi. In addition, almost no study on vector-borne pathogens in rodents has been done in Guangxi although diverse rodents are present locally. In this study, rodents were captured from probable infection sites of most of such patients to enhance the understanding of the genetic diversity of vector-borne bacteria and protozoan in rodents. The objective of this study was to reveal potential pathogens responsible for these patients.

## Materials and methods

### Ethics statement

This study was approved by the Ethics Committee of Chengde Medical University (No. 202004). All the rodents were treated in accordance with the “Rules for Implementation of Laboratory Animal Medicine” from the National Health Commission, China.

### Sample collection and DNA extraction

**Fig 1:**
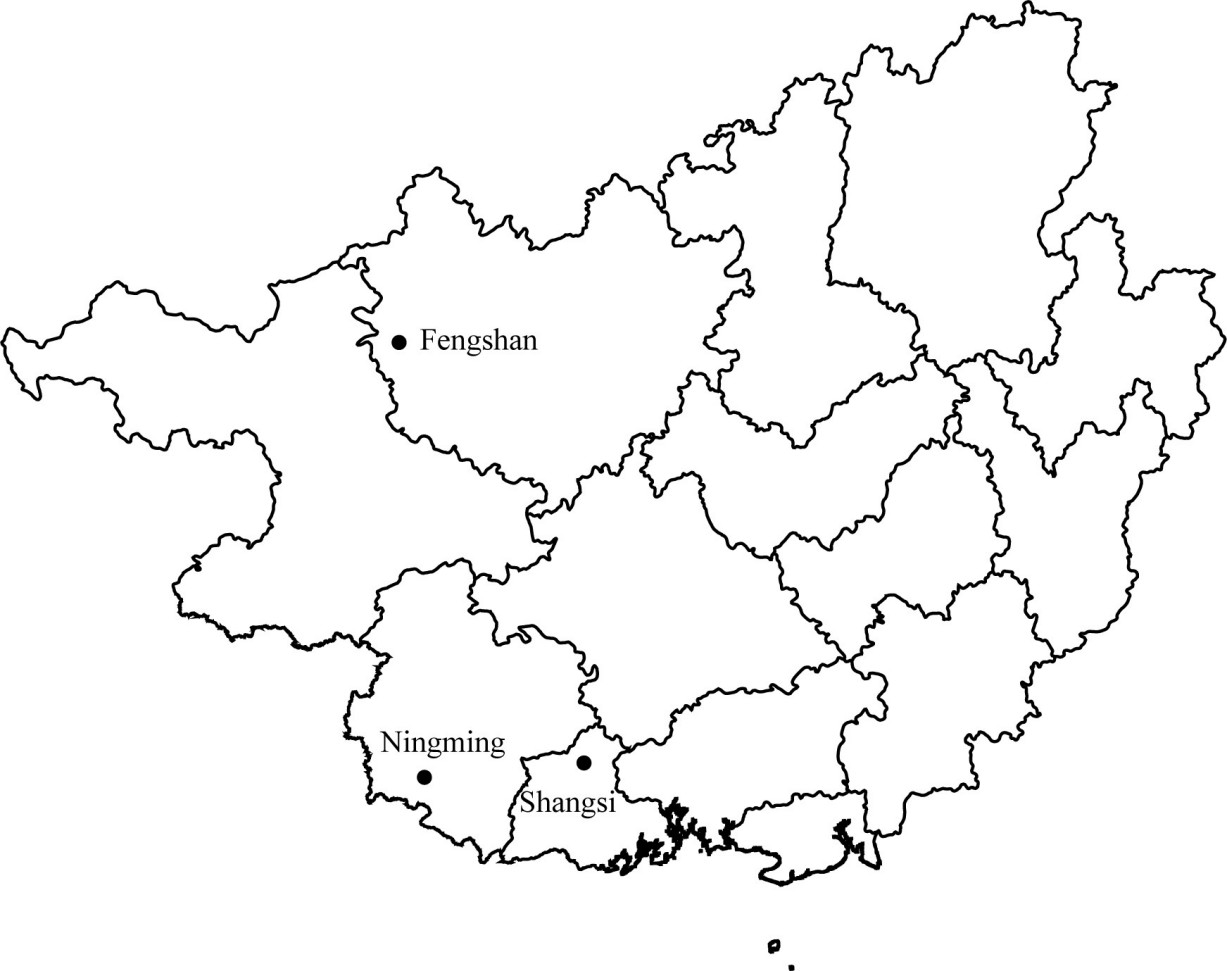
Geographical distribution of the rodent sampling sites (black dots) in Guangxi Province, China. The figure was generated using Rstudio. The source of the basemap shapefile was obtained from GADM (https://geodata.ucdavis.edu/gadm/gadm4.1/shp/gadm41_ZMB_shp.zip).

From May to October 2019, rodents were collected in wild environment around fields from Fengshan county of Hechi city, Ningming county of Chongzuo city, and Shangsi county of Fangchenggang city in Guangxi, China (Fig 1). The rodents were captured alive using baited cages with a treadle release mechanism (27×14×11). Two trapping sessions were conducted per month, and a total of two hundred cages were set up in each session. The trap line was set at the edge of the fields, and the cage traps were set at 10 m intervals. All the collected rodents were anesthetized with isofluorane before euthanasia to minimize suffering. The spleen tissue was aseptically collected after sacrifice. The rodent species were first identified based on morphological characteristics [27]. Rodents that tested positive for vector-borne microorganisms, approximately one-third of those that tested negative, and those with a small sample size were further confirmed at the species level by sequence analysis of the mitochondrial cytochrome b (mt-*cyt b*) gene [28]. Twenty-five milli grams of spleen tissue per sample was homogenized in 200 μL of TL Buffer (tissue lysis buffer). Total DNA was extracted using a Tissue DNA Kit (Omega, Norcross, GA, USA) and eluted in 80 μL of double-distilled water as per the manufacturer’s protocol in a fume hood in a separate laboratory room. The OD260/OD280 ratio of all the extracted DNA samples was 1.73–1.87. The extracted DNA was stored at -20°C before microorganism detection.

### Detection and molecular characterization of diverse microorganisms

The presence of microorganisms in rodents was identified by amplifying some marker genes using (semi-)nested PCR [12,21,29–32]. PrimeSTAR GXL Premix (Takara, Dalian, China) was used in all PCR reaction for the detection of microorganisms. Genera *Ehrlichia* and *Anaplasma* were screened using primer pairs of fD1/Eh-out2 and fD1/Eh-gs2, and Eh-out1/rp2 and Eh-gs1/rp2 targeting 16S rRNA gene [29,30]. “*Candidatus* N. mikurensis” was identified using primer pairs of CNM-out1/CNM-in2 and CNM-in1/CNM-R targeting *groEL* gene [21]. Primer pairs of bovis-gltA-F1/bovis-gltA-R and bovis-gltA-F2/bovis-gltA-R were used to detect *A*. *bovis* by amplifying the *gltA* gene [31]. Primer pairs of Abovis-groEL-F1/Abovis-groEL-R1 and Abovis-groEL-F2/Abovis-groEL-R2, designed in this study, were used to detect a novel lineage of *A*. *bovis* (identified from Australia) by amplifying the *groEL* gene. Primer pairs of HS1/HS6 and HS3/HSVR [33,34] were used to detect the genera *Anaplasm*a and *Ehrlichia* by amplifying the *groEL* gene. Primer pairs of Ehrlichia-1F/Ehrlichia-R and Ehrlichia-2F/Ehrlichia-R (designed in this study) were used to detect the genus *Ehrlichia* by amplifying the *gltA* gene. Primer pairs of CS7F2/HG1085R and F1b/AnaCS1076R were used to amplify the *gltA* gene of the genus *Anaplasma* [32]. Genus *Bartonella* was identified by amplifying both the *gltA* and *ftsz* genes. Primer pairs of Bar-gltA-F (Bar-gltA-FM)/Bar-gltA-R1 and Bar-gltA-F (Bar-gltA-FM)/Bar-gltA-R2 were used for *gltA* and Bar-ftsz-F1/Bar-ftsz-R (Bar-ftsz-RM) and Bar-ftsz-F2/Bar-ftsz-R (Bar-ftsz-RM) for *ftsz* gene, respectively [12]. Primer pairs of New-Babesia-F/New-Babesia-R1 and New-Babesia-F/New-Babesia-R2, designed in this study, were used to identify genera *Babesia* and *Hepatozoon* by amplifying18S rRNA gene. All the primer sequences are shown in Table 1.

**Table 1:** Primer sequences used in this study.

Pathogens	Target gene	round	Primer	Oligonucleotide sequences (5’- 3’)	Size	Tm	References
*Anaplasma* and *Ehrlichia*	*rrs*	first	fD1	AGAGTTTGATCCTGGCTCAG (+)	707 bp	55°C	29,30
			Eh-out2	AGTAYCGRACCAGATAGCCGC (-)			
		second	fD1	AGAGTTTGATCCTGGCTCAG (+)	639 bp	55°C	
			Eh-gs2	CTAGGAATTCCGCTATCCTCT (-)			
		first	Eh-out1	AACGAACGCTGGCGGCAAGC (+)	1,449 bp	55°C	
			rp2	ACGGCTACCTTGTDACGACTT (-)			
		second	Eh-gs1	TGCATAGGAATCTACCTAGTAG (+)	1,354 bp	55°C	
			rp2	ACGGCTACCTTGTDACGACTT (-)			
“*Candidatus* N. mikurensis”	*groEL*	first	CNM-out1	TGGCAAATGTAGTTGTAACAGG (+)	1,024 bp	50°C	21
			CNM-in2	GAAGAATTACTATCTACRCTACC (-)			
		second	CNM-in1	GCTATTAGTAAGCCTTATGGTAC (+)	631 bp	50°C	
			CNM-R	GYAGWGGTCTACCTGATCTTGCT (-)			This study
*A*. *bovis*	*gltA*	first	bovis-gltA-F1	TACATCWACWGTAAGAATGG (+)	360 bp	50°C	31
			bovis-gltA-R	TCWATGAAGTAYTCATCCT (-)			
		second	bovis-gltA-F2	ACWGTAAGAATGGTKGGCTC (+)	353 bp	50°C	
			bovis-gltA-R	TCWATGAAGTAYTCATCCT (-)			
	*groEL*	first	Abovis-groEL-F1	CTCTTATGTCNATGAGACG (+)	795 bp	50°C	This study
			Abovis-groEL-R1	TACGCTCYTTTACTTCYACT (-)			
		second	Abovis-groEL-F2	GTTGAAGAAGARGAAATAGC (+)	356 bp	50°C	
			Abovis-groEL-R2	CCTTCCACATCTTCAGCTAT (-)			
*Ehrlichia*	*gltA*	first	Ehrlichia-1F	CCAGGHTTTATGTCWACTGC (+)	1,092 bp	50°C	This study
			Ehrlichia-R	ACTGACGTGGACGACATATYT (-)			
		second	Ehrlichia-2F	TTTATGTCWACTGCTGCTTGT (+)	1,086 bp	50°C	
			Ehrlichia-R	ACTGACGTGGACGACATATYT (-)		50°C	
	*groEL*	first	HS1	CGYCAGTGGGCTGGTAATGAA (+)	1,412 bp	52°C	33,34
			HS6	CCWCCWGGTACWACACCTTC (-)			
		second	HS3	ATAGTYATGAAGGAGAGTGAT (+)	1,369 bp	55°C	
			HSVR	TCAACAGCAGCTCTAGTWG (-)			
*Anaplasma*	*gltA*	first	CS7F2	ATGRTAGAAAAWGCTGTTTT (+)	1,091 bp	52°C	32
			HG1085R	ACTATACCKGAGTAAAAGTC (-)			
		second	F1b	GAYCAYGARCARAATGCYTC (+)	416 bp	52°C	
			AnaCS1076	GAGTAAAAGTCGACRTTKGG (-)			
*Bartonella*	*gltA*	first	Bar-gltA-F/Bar-gltA-FM	TTACYTAYGAYCCYGGBTTTA (+) /GCHGATCAYGARCAAAATGC (+)	1,086 bp /526 bp	54°C	12
			Bar-gltA-R1	CYTCRATCATTTCTTTCCAYTG (-)			
		second	Bar-gltA-F/Bar-gltA-FM	TTACYTAYGAYCCYGGBTTTA (+) /GCHGATCAYGARCAAAATGC (+)	1,036 bp /476 bp	54°C	
			Bar-gltA-R2	GCAAAVAGAACMGTRAACAT (-)			
	*ftsz*	first	Bar-ftsz-F1	ATGACGATTAATCTGCATCG (+)	866 bp /581 bp	50°C	
			Bar-ftsz-R/Bar-ftsz-RM	TCTTCRCGRATACGATTRGC (-) /TAAAGHACTTGRTCAGCCAT (-)			
		second	Bar-ftsz-F2	ATTAATCTGCATCGGCCAGA (+)	860 bp /575 bp	50°C	
			Bar-ftsz-R/Bar-ftsz-RM	TCTTCRCGRATACGATTRGC (-) /TAAAGHACTTGRTCAGCCAT (-)			
*Babesia*	18S rRNA	first	Babesia-F	GTAATTCCAGCTCCAATAGC (+)	1,050 bp	50°C	This study
			Babesia-R1	ATAATTCACCGGATCACTCG (-)			
		second	Babesia-F	GTAATTCCAGCTCCAATAGC (+)	679 bp	50°C	
			Babesia-R2	ATTAASCAGACAAATCACTC (-)			

For the detection of microorganisms using (semi-)nested PCR, several rigorous measures were taken to prevent contamination, including filter tips used in each assay, each assay (the PCR mixture preparation, template addition, and agarose gel electrophoresis) performed in a fume hood in three separate rooms, and negative control using ddH_2_O as a template.

The PCR product of the expected size analyzed by denaturing gel electrophoresis was purified using the Takara MiniBEST Agarose Gel DNA Extraction Kit Version 4.0 (Takara, Dalian, China). The purified PCR product was sent to Shenggong Biotechnology Co., Ltd. for sequencing using the PCR primers. The PCR amplicons were sequenced with the ABI-PRISM Dye Termination Sequencing kit (Thermo Fisher Scientific, Waltham, MA) using an ABI 373-A genetic analyzer (Applied Biosystems, Carlsbad, CA).

### Sequence analysis

All the newly generated nucleotide sequences were edited using BioEdit [35], and then blasted against the nucleotide sequences deposited in the GenBank database (https://blast.ncbi.nlm.nih.gov) to determine the similarity. Nucleotide sequence identity was determined among sequences obtained in this study and reference sequences downloaded from the GenBank database using the MegAlign program in Lasergene [36]. The maximum likelihood (ML) tree was reconstructed using PhyML v3.2 [37] under the best-fit substitution model determined by MEGA 7.0 [38]. Bootstrap value determined with 1000 replicates was used to estimate the confidence values for each branch of the ML tree.

## Results

### Rodent sample collection

**Fig 2:**
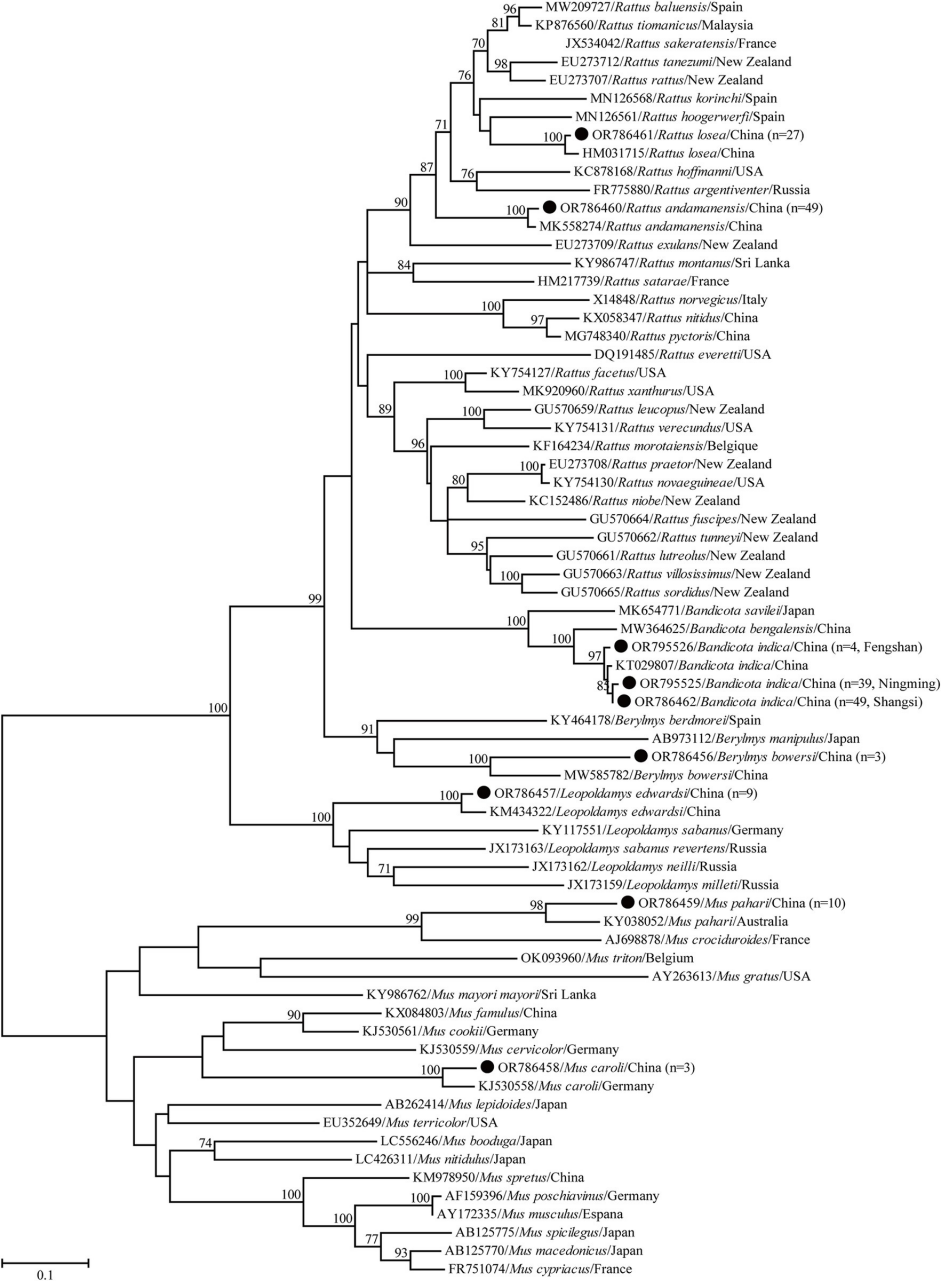
ML tree reconstructed based on the *cytb* gene sequences of rodent species. Bootstrap values were calculated with 1000 replicates and only >70% are shown. Sequences of rodent species determined herein are marked with black circle. One representative *cytb* gene sequence for each rodent species was used for the phylogenetic analysis.

A total of 192 rodents were collected in three sampling sites of Guangxi, China (Fig 1), and seven species belonging to five genera were tentatively identified based on the morphological characteristics. Consistently, the rodents captured in the current study were classified into seven clades in the phylogenetic tree based on the *cytb* gene (Fig 2), and these seven clades corresponded to seven rodent species, namely *Bandicota indica* (Greater Bandicoot Rat, n = 91), *Berylmys bowersi* (n = 3), *Leopoldamys edwardsi* (Edward’s long-tailed rat, n = 9), *Mus caroli* (n = 3), *Mus pahari* (Gairdner’s shrew-mouse, n = 10), *Rattus andamanensis* (n = 49), and *Rattus losea* (Lesser Rice-field Rat, n = 27) (S2 Table).

### Genetic and phylogenetic analysis of *Ehrlichia*

**Fig 3:**
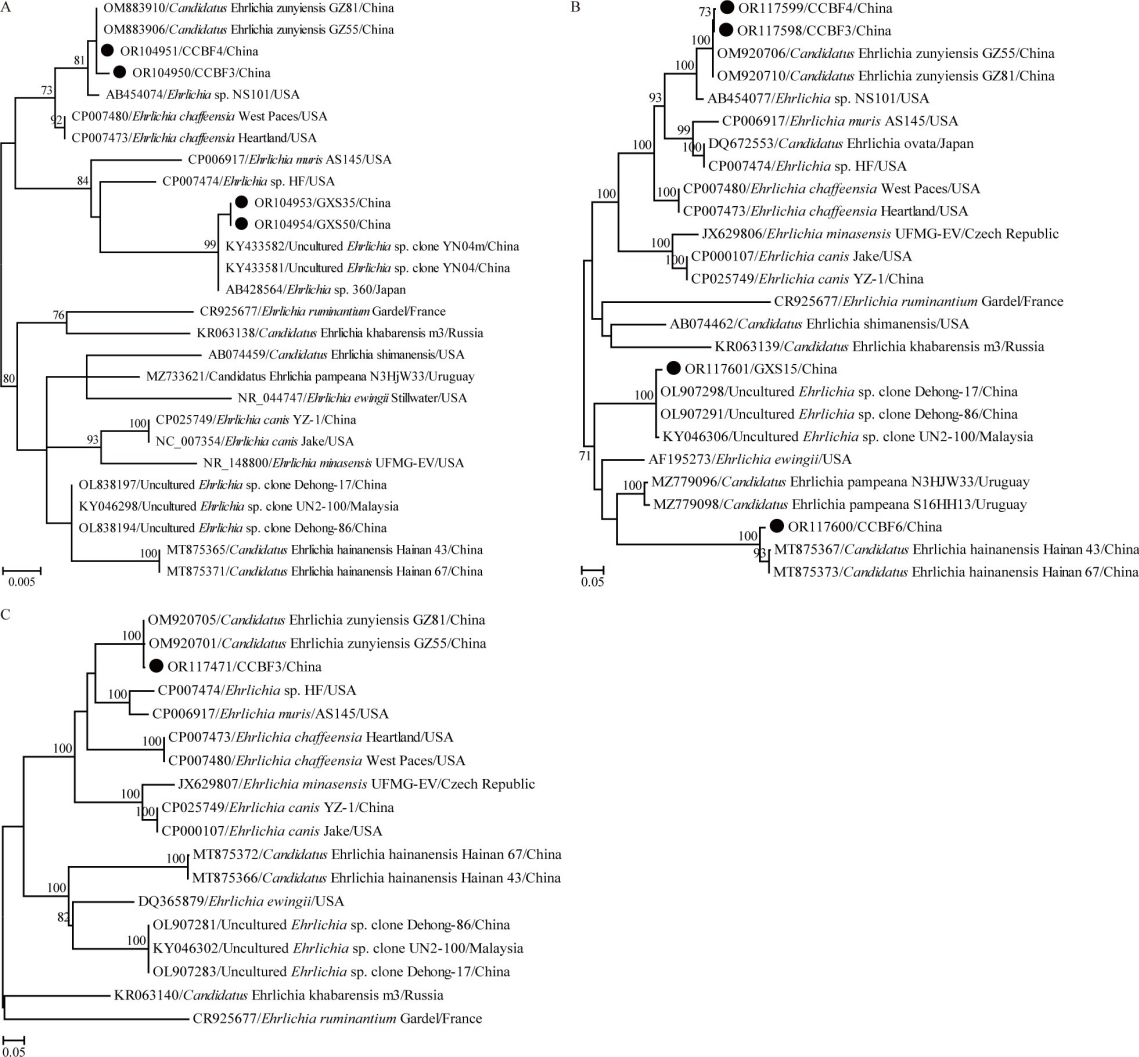
ML trees reconstructed based on the genus *Ehrlichia*. (A) ML tree based on partial 16S rRNA gene; (B) ML tree based on partial *groEL* gene; (C) ML tree based on partial *gltA* gene. The legend follows that of Fig 2.

Using primer pairs fD1/Eh-out2 and fD1/Eh-gs2, sample GXS19 tested positive for the genus *Ehrlichia* (Tables 2 and S2), and presented the highest nucleotide identity of 98.73% with some strains of *E*. *chaffeensis* for 16S rRNA gene (Query cover: 100%, E-value: 0.0, MN368552, NR_074500, CP007475–CP007480, CP007473 and CP000236). Furthermore, two partial 16S rRNA gene sequences were recovered by PCR with primer pairs Eh-out1/rp2 and Eh-gs1/rp2 (Table 2). All these two sequences (GXS35 and GXS50, S2 Table) shared the highest nucleotide identity of 99.8% with those of uncultured *Ehrlichia* sp. clone YN04m, uncultured *Ehrlichia* sp. clone YN04 and *Ehrlichia* sp. 360 (Query cover: 100%, E-value: 0.0), and all of them clustered together in the phylogenetic tree (Fig 3A). Unfortunately, we failed to get other genes whether the primers in the previous studies or designed in this study were applied in the PCR reactions.

**Table 2:** Detection of vector-borne microorganisms in wild rodents from Guangxi, China.

	Fengshan							Ningming	Shangsi
	*Rat*. *andamanensis*	*Rat*. *losea*	*Mus caroli*	*Mus pahari*	*Ber*. *bowersi*	*Leo*. *edwardsi*	*Ban*. *indica*	*Ban*. *indica*	*Ban*. *indica*
*A*. *bovis*	3/49^a^	0/27	0/3	1/10	0/3	0/9	0/14	0/38	1/49
*A*. *capra*	0/49	0/27	0/3	0/10	0/3	0/9	0/14	0/38	0/49
*A*. *ovis*	2/49	2/27	0/3	0/10	0/3	0/9	0/14	0/38	0/49
*A*. *phagocytophilum*	3/49	0/27	0/3	0/10	0/3	0/9	0/14	0/38	0/49
*Ca*. N. mikurensis	21/49	3/27	1/3	0/10	0/3	0/9	0/14	0/38	2/49
*Ca*. E. hainanensis	0/49	0/27	0/3	0/10	0/3	1/9	0/14	0/38	0/49
*Ca*. E. zunyiensis	0/49	0/27	0/3	0/10	0/3	2/9	0/14	0/38	0/49
uncultured *Ehrlichia* sp. 1 (*E*. *chaffeensis-*like)	1/49	0/27	0/3	0/10	0/3	0/9	0/14	0/38	0/49
uncultured *Ehrlichia* sp. 2 (uncultured *Ehrlichia* sp. clone YN04m)	2/49	0/27	0/3	0/10	0/3	0/9	0/14	0/38	0/49
uncultured *Ehrlichia* sp. 3 (uncultured *Ehrlichia* sp. clone Dehong-17)	1/49	0/27	0/3	0/10	0/3	0/9	0/14	0/38	0/49
*Bar*. *coopersplainsensis*	0/49	0/27	0/3	0/10	0/3	1/9	0/14	0/38	0/49
*Bar*. *tribocorum*	2/49	4/27	0/3	0/10	0/3	2/9	0/14	2/38	2/49
*Bar*. *rattimassiliensis*	0/49	2/27	0/3	0/10	0/3	0/9	0/14	0/38	0/49
*Bar*. *silvatica*	0/49	0/27	0/3	0/10	0/3	0/9	0/14	2/38	4/49
*Ca*. Bar. fengshanensis	0/49	3/27	0/3	0/10	0/3	0/9	0/14	0/38	0/49
*Ca*. Bar. shangsiensis	0/49	0/27	0/3	0/10	0/3	0/9	0/14	0/38	6/49
*Bab*. *microti*	3/49	0/27	0/3	2/10	0/3	0/9	0/14	0/38	0/49
*Hepatozoon* spp.	1/49	9/27	0/3	0/10	0/3	0/9	0/14	0/38	0/49

^a^ positive samples/total samples

Two species in the genus *Ehrlichia* were identified by PCR with the primer pairs for detection of “*Ca*. N. mikurensis” (Table 2). In the *groEL* gene tree (Fig 3B), “*Ca*. E. hainanensis” isolate CCBF6 presented the closest relationship with both “*Ca*. E. hainanensis” clones Hainan 67 and Hainan 43. Consistently, BLASTn showed that CCBF6 shared the highest nucleotide identity of 98.6% with both these two “*Ca*. E. hainanensis” isolates identified in *Niviventer fulvescens* from Hainan Province for the *groEL* gene. In addition to “*Ca*. E. hainanensis”, another uncultured *Ehrlichia* species was identified in sample GXS15 (Tables 2 and S2). BLASTn showed that its *groEL* gene sequence had the highest nucleotide identity of 99.5% with those of uncultured *Ehrlichia* sp. clone Dehong-17 and Dehong-86 in *Rhipicephalus microplus* collected from Yunnan of China, followed by uncultured *Ehrlichia* sp. clone UN2-100 in *Rhi*. *microplus* from Malaysia. In the *groEL* gene tree (Fig 3B), GXS15 clustered with the above-mentioned three *Ehrlichia* isolates.

In addition to the above-mentioned potential *Ehrlichia* species, two samples (CCBF3 and CCBF4) tested positive for “*Ca*. E. zunyiensis” using primer pairs Eh-out1/rp2 and Eh-gs1/rp2 (Tables 2 and S2). In addition, two partial *groEL* gene sequences were obtained using primer pairs HS1/HS6 and HS3/HSVR, and one partial *gltA* gene sequence was obtained using primer pairs Ehrlichia-1F/Ehrlichia-R and Ehrlichia-2F/Ehrlichia-R. These two isolates shared 100% nucleotide identity for partial 16S rRNA gene and 99.9% for *groEL* gene with each other. Furthermore, these two isolates had the highest nucleotide identity with known “*Ca*. E. zunyiensis” isolates, presenting 99.8% nucleotide identity for 16S rRNA gene, 99.8–99.9% nucleotide identities for *gltA* gene and 99.8–99.9% for *groEL* gene. Consistently, in the three trees based on 16S rRNA, *gltA* and *groEL* genes, these two isolates in this study clustered with the “*Ca*. E. zunyiensis” isolates identified in *Be*. *bowersi* collected from Zunyi City of Guizhou Province (Fig 3A–3C).

### Genetic and phylogenetic analysis of *Anaplasma*

**Fig 4:**
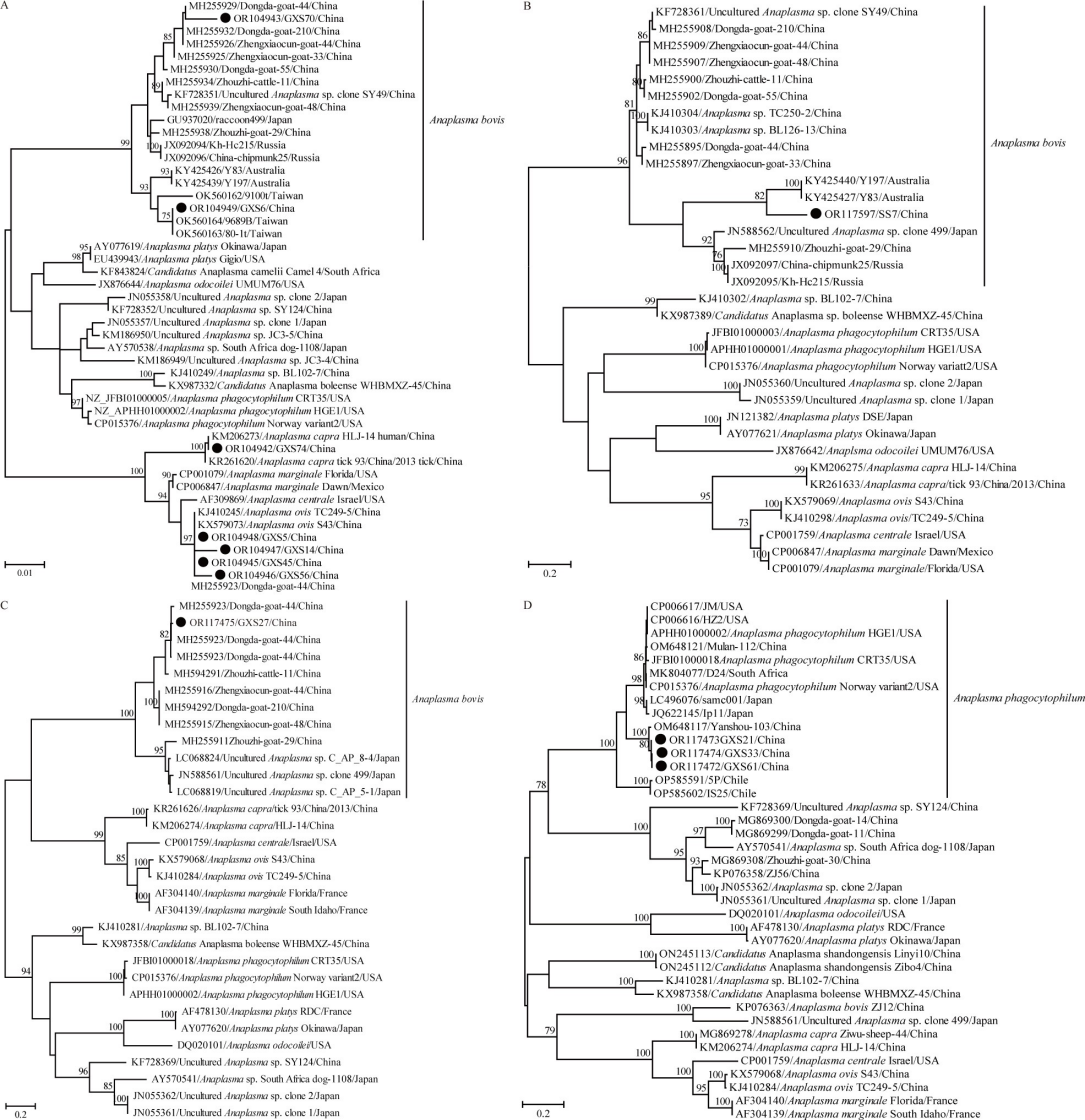
ML trees reconstructed based on the genus *Anaplasma*. (A) ML tree based on partial 16S rRNA gene; (B) ML tree based on partial *groEL* gene; (C) ML tree based on partial *gltA* gene of *Anaplasma bovis*; (D) ML tree based on partial *gltA* gene of *Anaplasma phagocytophilum*. The legend follows that of Fig 2.

Using primer pairs fD1/Eh-out2 and fD1/Eh-gs2, six samples tested positive for the genus *Anaplasma* (Tables 2 and S2). Of the six partial 16S rRNA gene sequences, BLASTn showed that four sequences (GXS5, GXS45, GXS14 and GXS56) had the highest nucleotide identity of 100% with some 16S rRNA gene sequences of *A*. *ovis* deposited in GenBank (Query cover: 100%, E-value: 0.0), one (GXS70) presented the highest nucleotide identity of 99.8% with that of *A*. *bovis* isolate Zhouzhi-cattle-10 (Query cover: 100%, E-value: 0.0), and the last (GXS74) shared the highest nucleotide identity of 100% with some 16S rRNA gene sequences of *A*. *capra* (Query cover: 100%, E-value: 0.0). Using primer pairs Eh-out1/rp2 and Eh-gs1/rp2, one partial 16S rRNA gene sequence was recovered from sample GXS6, which most closely resembled those of *A*. *bovis*. Interestingly, BLASTn showed that this sequence had 100% nucleotide identity with *A*. *bovis* isolate 9689B recovered from *Ban*. *indica* and *A*. *bovis* isolate 80-1t from *H*. *bandicota* parasitizing *Ban*. *indica* from Taiwan, 99.7% with *A*. *bovis* isolate 9100t from *Rhi*. *haemaphysaloides* parasitizing *Mus caroli* also from Taiwan, 99.6% with isolates from *Amblyomma triguttatum* from Australia, and 98.9–99.2% nucleotide identities with other *A*. *bovis* isolates. In the phylogenetic tree, all these seven isolates were classified into three clades, and corresponded to *A*. *capra*, *A*. *ovis* and *A*. *bovis*, respectively (Fig 4A). Especially, isolate GXS6 first clustered with isolates 9689B, 80-1t and 9100t from Taiwan, and then all of them formed a clade with isolates from Australia in the diversity of *A*. *bovis*.

Because of the absence of *gltA* and *groEL* genes of isolates 9689B, 80-1t and 9100t, primers based on *groEL* gene of isolates above-mentioned identified in Australia were designed. Surprisingly, using the primer pairs Abovis-groEL-F1/Abovis-groEL-R1 and Abovis-groEL-F2/Abovis-groEL-R2, one partial *groEL* gene sequence was amplified successfully from sample SS7 rather than from sample GXS6 (Tables 2 and S2). The partial *groEL* gene sequence showed the highest nucleotide identity of 91.4% (Query cover: 96%, E-value: 1e-111) with *A*. *bovis* isolates identified from Australia, followed by isolate Zhouzhi-goat-29 with 84.6% nucleotide identity (Query cover: 99%, E-value: 7e-80). However, it only presented less than 80% nucleotide identity with other *A*. *bovis* isolates both in and outside China. In the absence of a generally recognized cutoff value to identify a novel *Anaplasma* species, as well as the absence of other gene sequences, it is difficult to determine whether it represents a new species or just a variant of *A*. *bovis*. In the phylogenetic tree, this partial *groEL* gene sequence was closely related to those isolates recovered from *Amblyomma triguttatum* in Australia (Fig 4B). Additionally, sample GXS27 tested positive for *A*. *bovis* using prime pairs bovis-gltA-F1/bovis-gltA-R and bovis-gltA-F2/bovis-gltA-R (Tables 2 and S2). In the *gltA* gene tree, this *A*. *bovis* isolate fell into the diversity of lineage 1 of Guo et al. [31] (Fig 4C). The nucleotide sequence identity between the *gltA* sequence in this study and those belonging to lineage 1 ranged from 94.1% to 99.4%. We also tried to obtain the 16S rRNA and *groEL* sequences using the primers listed above and in the previously study but failed.

Furthermore, *Anaplasma* spp. was identified using primer pairs CS7F2/HG1085R and F1b/AnaCS1076R. As a result, three partial *gltA* gene sequences were recovered from samples GXS21, GXS33 and GXS61 (Tables 2 and S2), and all of them showed 99.5–99.7% nucleotide identities with each other. BLASTn showed that all these three *gltA* gene sequences shared the highest nucleotide identities of 98.4–98.7% with that of *A*. *phagocytophilum* isolate Yanshou-103 identified in *Haemaphysalis longicornis* tick from Heilongjiang Province (Query cover: 100%, E-value: 0.0), followed by *A*. *phagocytophilum* isolates with approximately 85.1–88.4% nucleotide identities. In the phylogenetic tree, these three isolates in this study were closely related to isolate Yanshou-103 and clustered together, and separate from other *A*. *phagocytophilum* isolates (Fig 4D). Although the primer pairs targeting the *groEL* gene was designed based on the corresponding sequences of isolate Yanshou-103 and other *A*. *phagocytophilum* isolates, we failed to obtain the *groEL* gene from these three positive samples.

### Genetic and phylogenetic analysis of *Candidatus* Neoehrlichia

**Fig 5:**
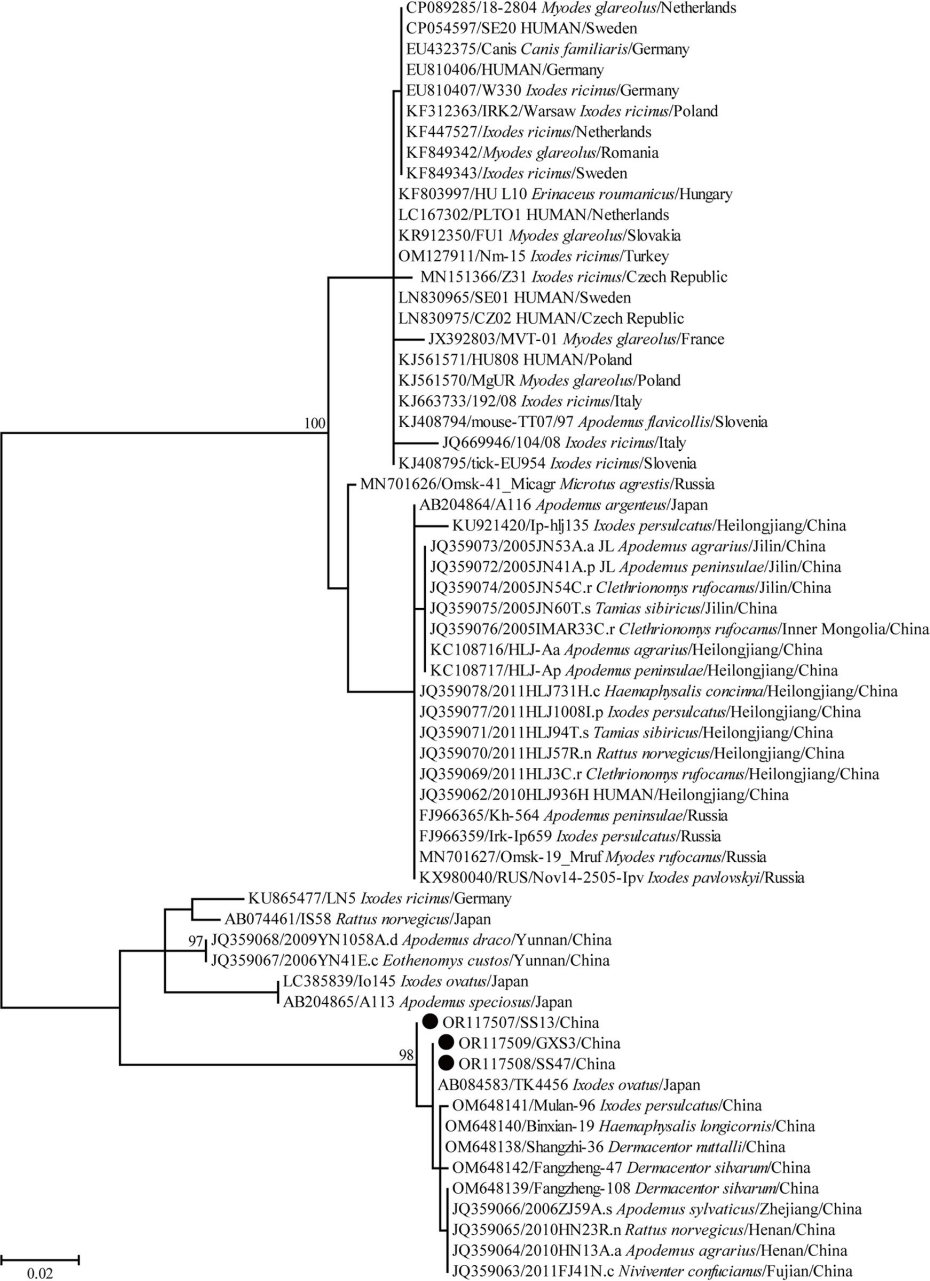
ML trees reconstructed based on “*Candidatus* Neoehrlichia mikurensis”. Three representative *groEL* gene sequences were used for the phylogenetic analysis. The legend follows that of Fig 2.

BLASTn showed that 27 rodent samples tested positive for “*Ca*. N. mikurensis” (Tables 2 and S2). All these 27 partial *groEL* gene sequences were closely related to each other, and presented 99.8–100% nucleotide identities. Interestingly, all these isolates clustered with five isolates (Mulan-96, Fangzheng-108, Binxian-19, Shangzhi-36 and Fangzheng-47) from Heilongjiang Province, four (2011FJ41N.c, 2010HN13A.a, 2010HN23R.n and 2006ZJ59A.s ZJ) from Southeast China and one (TK4456) from Japan, and formed a separate clade in the phylogenetic tree (Fig 5), rather than with isolates from Yunnan Province neighboring Guangxi. All the isolates in this clade shared 99.3–100% nucleotide identities.

### Genetic and phylogenetic analysis of *Bartonella*

**Fig 6:**
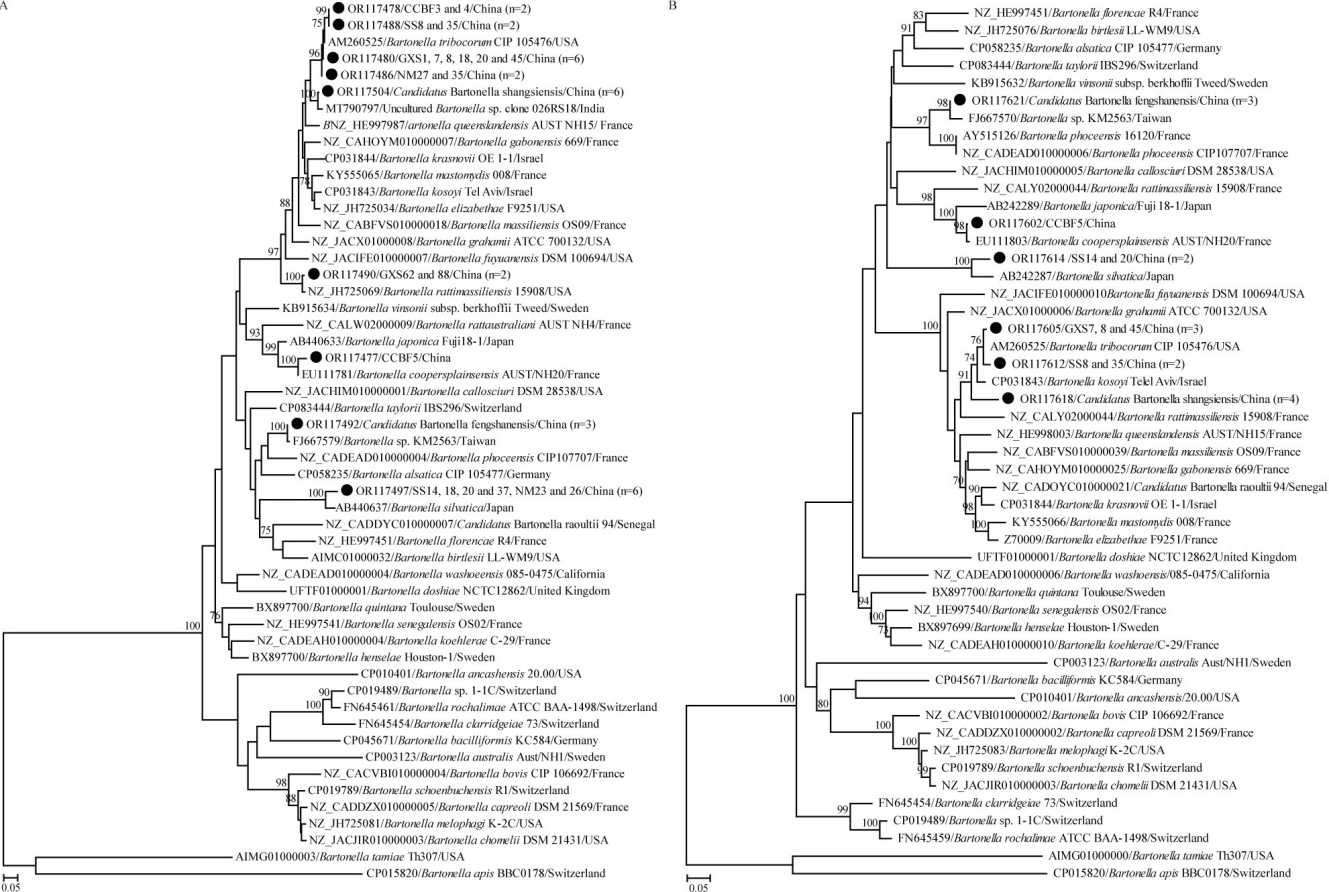
ML tree reconstructed based on the genus *Bartonella*. (A) ML tree based on partial *ftsz* gene; (B) ML tree based on partial *gltA* gene. One representative sequence of *ftsz* and *gltA* genes for each *Bartonella* species was used for the phylogenetic analysis. The legend follows that of Fig 2.

Detection of *Bartonella* spp. targeting both the *ftsz* and *gltA* genes revealed that 30 samples were positive. BLASTn showed that four validated *Bartonella* species were identified, including *Bar*. *coopersplainsensis*, *Bar*. *tribocorum*, *Bar*. *rattimassiliensis*, and *Bar*. *silvatica* (Tables 2 and S2). In addition, a *Bartonella* species closely related to *Bartonella* sp. KM2563 was detected, and all three isolates shared the highest nucleotide identities of 99.8% and 99.3–99.6% with *Bartonella* sp. KM2563 for the *ftsz* and *gltA* genes, respectively, followed by *Bar*. *phoceensis*. Furthermore, another *Bartonella* species was detected (Tables 2 and S2), which was closely related to uncultured *Bartonella* sp. clone 026RS18FTSZ in *Ra*. *satarae* from India, followed by *Bar*. *tribocorum*. Six *ftsz* gene sequences were recovered from all the positive samples, and they shared 99.6–100% nucleotide identities with each other, and 99.2–99.4% with uncultured *Bartonella* sp. clone 026RS18FTSZ and 97.3% with *Bar*. *tribocorum*. Moreover, two *gltA* gene sequences were recovered, and they shared 99.5–99.9% nucleotide identities to each other and 96.3–96.4% with *Bar*. *tribocorum* due to an absence of the *gltA* gene of uncultured *Bartonella* sp. clone 026RS18FTSZ. As shown in Fig 6, the phylogenetic trees based on the *ftsZ* and *gltA* genes showed a similar topology, and the newly identified isolates in this study clustered with the corresponding isolates of *Bar*. *coopersplainsensis*, *Bar*. *tribocorum*, *Bar*. *rattimassiliensis* and *Bar*. *silvatica*, respectively. Interestingly, the three isolates closely related to *Bartonella* sp. KM2563 was determined in this study and *Bartonella* sp. KM2563 formed a separate clade in both *ftsZ* and *gltA* genes trees. Both nucleotide and phylogenetic analyses suggested that they represent a novel species, and we name it “*Candidatus* Bartonella fengshanensis” according to the site where the rodents were collected. As for another six isolates closely related to uncultured *Bartonella* sp. clone 026RS18FTSZ, all of them first clustered with uncultured *Bartonella* sp. clone 026RS18FTSZ, and then with *Bar*. *tribocorum* in the *ftsZ* tree. On the contrary, these isolates formed a distinct clade and were separate from *Bar*. *tribocorum* in the *gltA* tree. Therefore, they represent another novel species although it was closely related to *Bar*. *tribocorum*, and we name it as “*Candidatus* Bartonella shangsiensis” according to the site where the rodents were collected.

### Genetic and phylogenetic analysis of protozoan parasites

**Fig 7:**
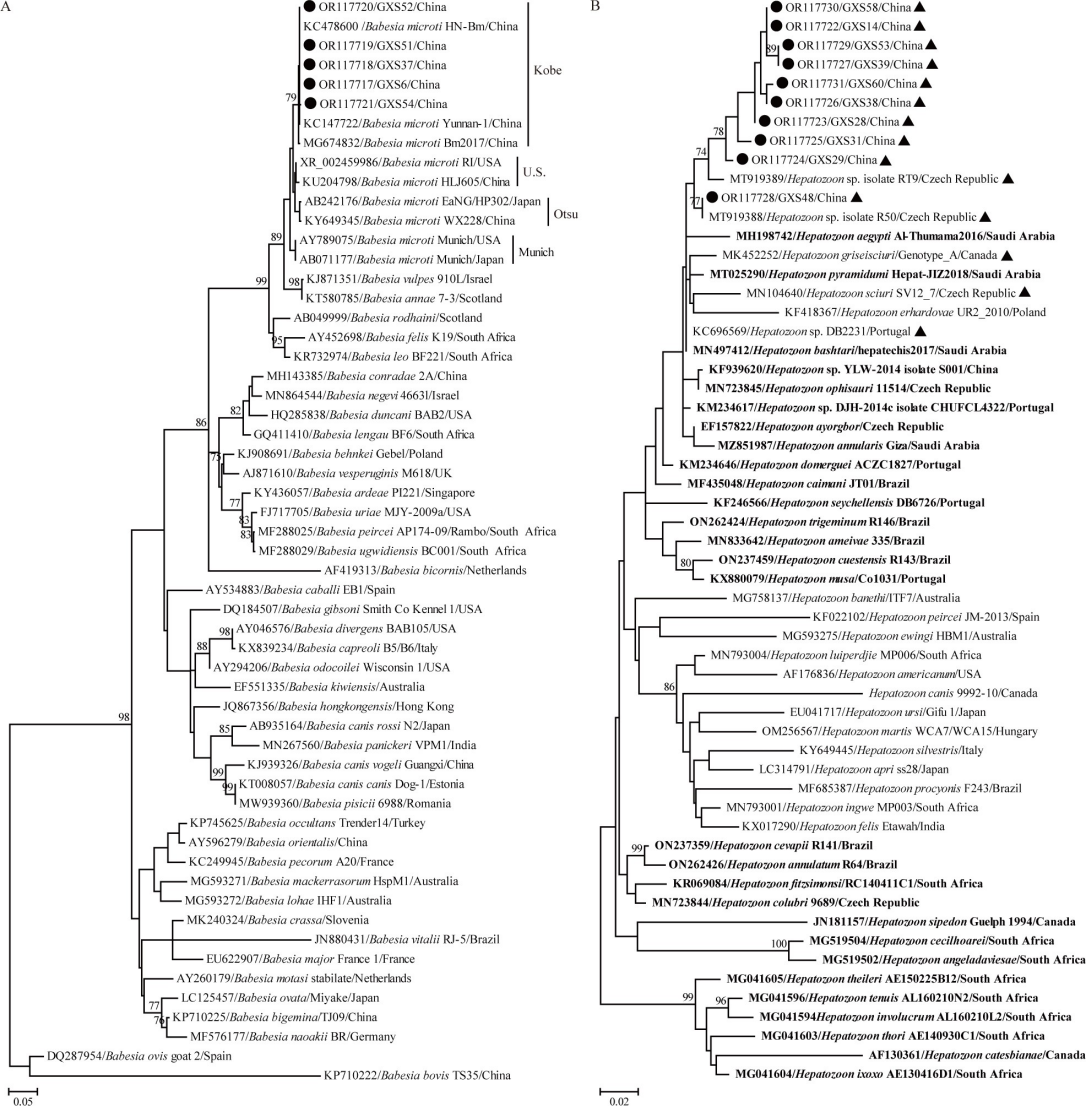
ML tree reconstructed based on partial 18S rRNA gene sequences of protozoan. (A) ML tree based on partial 18S rRNA gene sequences of the genus *Babesia*; (B) ML tree based on partial 18S rRNA gene sequences of the genus *Hepatozoon*. The legend follows that of Fig 2. The sequences marked with black triangle were identified in rodents, and those shown in bold were recovered from reptiles, including snakes.

By amplifying the partial 18S rRNA gene, 15 rodent samples tested positive protozoan parasites. BLASTn showed that five isolates belonged to *Bab*. *microti* and other 10 belonged to the genus *Hepatozoon* (Tables 2 and S2). The five partial 18S rRNA gene sequences of *Bab*. *microti* shared 99.9–100% nucleotide identities to each other and 98.1–100% with corresponding sequences of other known *Bab*. *microti* isolates. The 10 partial 18S rRNA gene sequences of *Hepatozoon* spp. shared 98.2–100% nucleotide identities with each other. Furthermore, GXS48 shared the highest identity of 100% with *Hepatozoon* sp. isolates R50 and R38. Of another nine, six showed the highest identities of 98.5–98.9% with *Hepatozoon* sp. isolate RT9 and the remaining three showed the highest identities of 98.8–98.9% with *Hepatozoon* sp. DJH-2014c, respectively. For *Bab*. *microti*, all five isolates determined in this study fell into the diversity of *Bab*. *microti*, and clustered with the Kobe-type isolates, a zoonotic genotype (Fig 7A). In the phylogenetic tree, GXS48 clustered together with *Hepatozoon* sp. isolates R50 and formed a distinct clade. Although clustering together, another nine isolates formed six distinct clades, suggesting potential novel *Hepatozoon* species (Fig 7B). Interestingly, *Hepatozoon* species associated with rodents and reptiles from different parts of the world formed a large clade in the phylogenetic tree based on 18S rRNA gene, and those identified in this study fell into the diversity of this clade.

### Co-infection of diverse microorganisms

Dual infection was observed between different microorganisms (S2 Table). Specifically, dual infection was identified between *A*. *bovis* and *Bar*. *tribocorum* in SS7, “*Ca*. E. zunyiensis” and *Bar*. *tribocorum* in CCBF3 and CCBF4, *A*. *bovis* and *Bab*. *microti* in GXS6, uncultured *Ehrlichia* sp. clone YN04m and “*Ca*. N. mikurensis” in GXS35, “*Ca*. Bar. fengshanensis” and “*Ca*. N. mikurensis” in GXS63, *A*. *phagocytophilum* and “*Ca*. N. mikurensis” in GXS21, and “*Ca*. Bar. fengshanensis” and “*Ca*. N. mikurensis” in GXS64 were identified. Interestingly, co-infection between “*Ca*. N. mikurensis” and other microorganisms is the most common type.

## Discussion

In this study, a molecular survey of vector-borne pathogens in field rodents was performed, and the results showed that four *Anaplasma*, five *Ehrlichia*, “*Ca*. N. mikurensis”, six *Bartonella*, *Bab*. *Microti* and diverse *Hepatozoon* were identified in six rodent species collected from three sampling sites in Guangxi Province. In addition, *A*. *capra*, *A*. *ovis* and *E*. *chaffeensis*-like, as well as several potential new *Hepatozoon* species, were identified although more evidences are needed, which is the limitation of this study. Of them, six, including *A*. *capra*, *A*. *phagocytophilum*, “*Ca*. N. mikurensis”, *Bar*. *rattimassiliensis*, *Bar*. *tribocorum* and *Bab*. *microti* are zoonotic pathogens in addition to *A*. *bovis* and *A*. *ovis* with zoonotic potential. Hence, our results greatly contribute to revealing the species diversity of rodent-borne bacteria and protozoan circulating in this area, especially those pathogenic to humans.

As the natural hosts of many *Anaplasma* and *Ehrlichia* species, rodents have been reported to harbor *A*. *phagocytophilum*, *A*. *bovis*, *A*. *ovis* [39], *E*. *chaffeensis*, *E*. *muris*, “*Ca*. E. hainanensis” [26], “*Ca*. E. zunyiensis” [25], “*Ca*. E. khabarensis”, and “*Ca*. E. extremiorentalis” (AY584851). In this study, sequence analysis showed that four *Anaplasma* and five *Ehrlichia* were detected in rodents, respectively. Interestingly, this is the first evidence of *A*. *capra* infection in rodents although further evidence is needed. *Anaplasma phagocytophilum* is distributed worldwide, and presents extensive genetic diversity. In this study, three isolates, GXS21, GXS33, and GXS61, were closely related to isolate Yanshou-103 [40], and formed a distinct clade in the phylogenetic tree, suggesting a novel lineage of *A*. *phagocytophilum*. Interestingly, these three isolates were identified in Guangxi in Southwest China; while isolate Yanshou-103 was identified in Heilongjiang in Northeast China. Hence, we propose a conjecture that this novel lineage of *A*. *phagocytophilum* may be present in other parts of China. For *A*. *bovis*, except known lineage circulating in mainland China, one 16S rRNA gene sequence presenting the closest relationship with isolates from Taiwan, and another *groEL* gene showing the highest nucleotide identity of 91.4% with isolates from Australia [41], were generated in this study. In the absence of a generally recognized cutoff value to identify a novel *Anaplasma* species, we temporarily suppose that they may be variants of *A*. *bovis* based on the phylogenetic analysis. However, these two sequences were amplified from two rodent samples from two different sampling sites, so we cannot determine whether these two sequences are from the same lineage of *A*. *bovis*. But anyway, there must be at least one novel lineage of *A*. *bovis* circulating locally. Previous studies demonstrated that “*Ca*. N. mikurensis” was widely distributed in rodents in and outside China, and its genetic diversity was correlated with the geographic origin [21]. In 2023, “*Ca*. N. mikurensis” isolates identified from northeastern China closely related to those from southeastern China were identified in ticks [40]. In this study, “*Ca*. N. mikurensis” isolates in this lineage were also identified in rodents, indicating its wide distribution.

As emerging zoonotic causative agents, most members of the genus *Bartonella* are hosted by diverse rodent species, with the prevalence reaching up to more than 50% in the field [10,11,22]. Of rodent-associated species, eight are regarded as human pathogens causing bartonellosis with a variety of clinical features, namely, *Bar*. *doshiae*, *Bar*. *elizabethae*, *Bar*.*grahamii*, *Bar*. *rattimassiliensis*, *Bar*. *rochalimae*, *Bar*. *tribocorum*, *Bar*. *vinsonii*, and *Bar*. *washoensis*, and all have been identified in China [12]. Based on the genetic and phylogenetic analysis, four validated *Bartonella* species, including *Bar*. *coopersplainsensis*, *Bar*. *tribocorum*, *Bar*. *rattimassiliensis*, and *Bar*. *silvatica*, were identified in four rodent species. Notably, *Bar*. *tribocorum* are considered to be zoonotic pathogens in recent years [42]. In addition, two potential novel species were detected in *Ra*. *losea* and *Ban*. *indica*, respectively. Specifically, one was most closely related to *Bar*. *phoceensis*, with approximate 95.5% nucleotide identities for *gltA* gene. Based on the criteria suggested by La Scola et al. [43] for *Bartonella* targeting the *gltA* gene (the *gltA* fragment shares <96.0% sequence similarity with validated species), this *Bar*. *phoceensis*-like species can be attributed to a novel species, named “*Ca*. Bartonella fengshanensis”. For another species, it had the closest relationship with *Bar*. *tribocorum* (96.4% nucleotide identity for *gltA* gene), which doesn’t fit the criteria suggested by La Scola et al [43]. However, *Bar*. *tribocorum* and *Bar*. *kosoyi* are known as two distinct species although they share 98.6% nucleotide identity for *gltA* gene. Combination the fact that less than 4% genetic divergence between two known *Bartonella* species and the position of *Bar*. *tribocorum*-like on the *gltA* tree, we still consider it as a novel *Bartonella* species, named “*Ca*. Bartonella shangsiensis”. The human-pathogenic *Bar*. *tribocorum* and two presumably novel *Bartonella* species infections in rodents call for more robust surveillance studies to reveal the prevalence of *Bartonella* in rodents, even in humans.

Of the *Babesia* species pathogenic to humans, *Bab*. *microti*, *Bab*. *venatorum*, and *Bab*. *divergens* have been identified in patients and cause babesiosis with atypical clinical systems in China [44]. Besides, *Bab*. *crassa*-like and *Babesia*. sp. XXB/Hangzhou causing sporadic cases have also been reported [45,46]. To date, only *Bab*. *microti* was detected in rodents in Yunnan, Beijing, Zhejiang, Fujian and Henan Provinces, although the above-mentioned pathogens were detected in ticks on a much wider geographical scale in China [23,24,47–50]. In Guangxi, *Bab*. *microti* has been identified in patients, ticks (*Rhi*. *sanguineus*), and farmed *Macaca fascicularis* in Nanning city [51–53]. Consistently, five *Bab*. *microti* isolates were identified in *Mus pahari* and *Ra*. *andamanensis* collected from Fengshan of Hechi city in the current study, indicating its wide geographical distribution in Guangxi. In addition, all five *Bab*. *microti* isolates belong to Kobe-type with zoonotic, therefore, surveillance focusing on human cases should be performed in the future study.

*Hepatozoon* spp. are important veterinary pathogens and their pathogenicity to humans is unclear although *Hepatozoon* sp. detected in humans from Russia has been reported [54]. Rodents are regarded as natural intermediate and potential paratenic hosts of some *Hepatozoon* species [55,56]. Furthermore, these agents have been identified in diverse rodent species around the world [20]. In China, *Hepatozoon* spp. have been identified in snakes [57,58], dogs [59], cats (GenBank: OM714911), and one rodent species, namely, *Rhombomys opimus* [19]. Therefore, the information on *Hepatozoon* spp. in rodents is scarce in China. In this study, ten *Hepatozoon* isolates were identified in *Mus pahari* and *Ra*. *andamanensis*. This is the first report of *Hepatozoon* spp. infections in these two rodents, expanding the rodent range. Considering the nucleotide identity among them and their position in the phylogenetic tree, nine isolates may belong to different potential novel species. In addition, the most related sequences in the phylogenetic tree were from rodents and snakes, hence, we put forward a hypothesis that *Hepatozoon* species can be transmitted from rodents as the paratenic host to reptiles, consistent with a previous study [20].

Several limitations existed in this study. First, an inadequate representation of rodent species existed in two sampling sites in Ningmign and Shangsi, respectively, resulting in an incomplete depiction of the vector-borne pathogens in wild rodents. Second, only one gene or partial gene sequences were obtained for some pathogens, affecting our understanding of their genetic characteristics. Third, the age or gender of the captured rodents was not recorded; therefore, the assessment of the prevalence of each pathogen across age groups or sexes of rodents could not be determined. Forth, blood-sucking vectors on the rodents were not collected in this study; therefore, it is uncertain whether there is consistency between the pathogens carried by rodents and those carried by arthropods on rodents.

## Conclusion

In sum, a wide variety of bacterial and protozoan microorganisms were identified in rodents from Guangxi Province, China. Of them, six are human pathogens, including one *Bartonella*, one *Babesia*, “*Ca*. N. mikurensis”, and three *Anaplasma*. In addition, potential novel *Bartonella* species and diverse uncultured *Hepatozoon* clones were identified, contributing to a better understanding of the genetic diversity of rodent-associated *Bartonella* and *Hepatozoon*, respectively. Our findings underscore the potential risk of transmission to humans and emphasize the need for enhanced surveillance of these causative agents in human populations. Furthermore, our results offer valuable insights to mitigate the public health risk posed by the causative agents identified in this study.

## Supporting information

S1 Table GenBank accession numbers of sequences obtained in this study. (DOCX)

S2 Table Pathogens detected in rodents from Guangxi, China. (DOCX)

## References

[1] Meerburg BG, Singleton GR, Kijlstra A. Rodent-borne diseases and their risks for public health. Crit Rev Microbiol. 2009; 35(3): 221–270. doi: 10.1080/10408410902989837 19548807

[2] Drancourt M, Houhamdi L, Raoult D. *Yersinia pestis* as a telluric, human ectoparasite-borne organism. Lancet Infect Dis. 2006; 6(4): 234–241. doi: 10.1016/S1473-3099(06)70438-8 16554248

[3] Kabwe E, Davidyuk Y, Shamsutdinov A, Garanina E, Martynova E, Kitaeva K, et al. Emerging Zoonotic Pathogens. Pathogens. 2020; 9(9): 775. doi: 10.3390/pathogens9090775 32971887 PMC7558059

[4] Brisse ME, Ly H. Hemorrhagic Fever-Causing Arenaviruses: Lethal Pathogens and Potent Immune Suppressors. Front Immunol. 2019; 10: 372. doi: 10.3389/fimmu.2019.00372 30918506 PMC6424867

[5] Carvalho CL, Lopes de Carvalho I, Zé-Zé L, Núncio MS, Duarte EL. Tularaemia: a challenging zoonosis. Comp Immunol Microbiol Infect Dis. 2014; 37(2): 85–96. doi: 10.1016/j.cimid.2014.01.002 24480622 PMC7124367

[6] Mohd Zain SN, Amdan SA, Braima KA, Abdul-Aziz NM, Wilson JJ, Sithambaran P, et al. Ectoparasites of murids in peninsular Malaysia and their associated diseases. Parasit Vectors. 2015; 8: 254. doi: 10.1186/s13071-015-0850-1 25924677 PMC4422404

[7] Darby AC, Cho NH, Fuxelius HH, Westberg J, Andersson SG. Intracellular pathogens go extreme: genome evolution in the Rickettsiales. Trends Genet. 2007; 23(10): 511–520. doi: 10.1016/j.tig.2007.08.002 17822801

[8] Merhe V, Angelakis E, Socolovschi C, Raoult D. Genotyping, evolution and epidemiological findings of *Rickettsia* species. Infect Genet Evol. 2014; 25: 122–37. doi: 10.1016/j.meegid.2014.03.014 24662440

[9] Rar V, Golovljova I. *Anaplasma*, *Ehrlichia*, and "*Candidatus* Neoehrlichia" bacteria: pathogenicity, biodiversity, and molecular genetic characteristics, a review. Infect Genet Evol. 2011; 11(8): 1842–1861. doi: 10.1016/j.meegid.2011.09.019 21983560

[10] Okaro U, Addisu A, Casanas B, Anderson B. *Bartonella* Species, an Emerging Cause of Blood-Culture-Negative Endocarditis. Clin Microbiol Rev. 2017; 30(3): 709–746. doi: 10.1128/CMR.00013-17 28490579 PMC5475225

[11] Krügel M, Król N, Kempf VAJ, Pfeffer M, Obiegala A. Emerging rodent-associated *Bartonella*: a threat for human health? Parasit Vectors. 2022; 15(1): 113. doi: 10.1186/s13071-022-05162-5 35361285 PMC8969336

[12] Jian R, Ren Q, Xue J, Xie GC, Wang J, Chen GQ, et al. Genetic diversity of *Bartonella* infection in residential and field rodents in Hebei, China. Front Microbiol. 2022; 13: 1039665. doi: 10.3389/fmicb.2022.1039665 36504836 PMC9732461

[13] Schnittger L, Rodriguez AE, Florin-Christensen M, Morrison DA. Babesia: a world emerging. Infect Genet Evol. 2012; 12(8): 1788–1809. doi: 10.1016/j.meegid.2012.07.004 22871652

[14] Vannier E, Krause PJ. Human babesiosis. N Engl J Med. 2012; 366(25): 2397–2407. doi: 10.1056/NEJMra1202018 22716978

[15] Barta JR. Molecular approaches for inferring evolutionary relationships among protistan parasites. Vet Parasitol. 2001; 101(3–4): 175–186. doi: 10.1016/s0304-4017(01)00564-7 11707295

[16] Baneth G. Perspectives on canine and feline hepatozoonosis. Vet Parasitol. 2011; 181(1): 3–11. doi: 10.1016/j.vetpar.2011.04.015 21620568

[17] Smith TG. The genus *Hepatozoon* (Apicomplexa: Adeleina). J Parasitol. 1996; 82(4): 565–585. 8691364

[18] Sloboda M, Kamler M, Bulantová J, Votýpka J, Modrý D. Rodents as intermediate hosts of *Hepatozoon ayorgbor* (Apicomplexa: Adeleina: Hepatozoidae) from the African ball python, *Python regius*. Folia Parasitol. 2008; 55(1): 13–16. doi: 10.14411/fp.2008.003 18578163

[19] Ji N, Chen X, Liu G, Zhao S, Tan W, Liu G, et al. *Theileria*, *Hepatozoon* and *Taenia* infection in great gerbils (*Rhombomys opimus*) in northwestern China. Int J Parasitol Parasites Wildl. 2021; 15: 79–86. doi: 10.1016/j.ijppaw.2021.04.002 33996439 PMC8099453

[20] Weck BC, Serpa MCA, Ramos VN, Luz HR, Costa FB, Ramirez DG, et al. Novel genotypes of *Hepatozoon* spp. in small mammals, Brazil. Parasit Vectors. 2022; 15(1): 87. doi: 10.1186/s13071-022-05216-8 35292086 PMC8922722

[21] Li H, Jiang J, Tang F, Sun Y, Li Z, Zhang W, et al. Wide distribution and genetic diversity of "*Candidatus* Neoehrlichia mikurensis" in rodents from China. Appl Environ Microbiol. 2013; 79(3): 1024–1027. doi: 10.1128/AEM.02917-12 23183973 PMC3568564

[22] Li DM, Hou Y, Song XP, Fu YQ, Li GC, Li M, et al. High prevalence and genetic heterogeneity of rodent-borne *Bartonella* species on Heixiazi Island, China. Appl Environ Microbiol. 2015; 81(23): 7981–7992. doi: 10.1128/AEM.02041-15 26362983 PMC4651081

[23] Gao ZH, Huang TH, Jiang BG, Jia N, Liu ZX, Shao ZT, et al. Wide Distribution and Genetic Diversity of *Babesia microti* in Small Mammals from Yunnan Province, Southwestern China. PLoS Negl Trop Dis. 2017; 11(10): e0005898. doi: 10.1371/journal.pntd.0005898 29059184 PMC5681298

[24] Wei CY, Wang XM, Wang ZS, Wang ZH, Guan ZZ, Zhang LH, et al. High prevalence of *Babesia microti* in small mammals in Beijing. Infect Dis Poverty. 2020; 9(1): 155. doi: 10.1186/s40249-020-00775-3 33176879 PMC7661193

[25] Lu M, Tang G, Ren Z, Zhang J, Wang W, Qin X, et al. *Ehrlichia*, *Coxiella* and *Bartonella* infections in rodents from Guizhou Province, Southwest China. Ticks Tick Borne Dis. 2022a; 13(5): 101974. doi: 10.1016/j.ttbdis.2022.101974 35662068

[26] Zhao GP, Wang YX, Fan ZW, Ji Y, Liu MJ, Zhang WH, et al. Mapping ticks and tick-borne pathogens in China. Nat Commun. 2021; 12(1): 1075. doi: 10.1038/s41467-021-21375-1 33597544 PMC7889899

[27] Wang YX. A complete checklist of mammal species and subspecies in China—a taxonomic and geographic reference. Beijing: China Forestry Publishing House. 2003 (in Chinese).

[28] Guo WP, Lin XD, Wang W, Tian JH, Cong ML, Zhang HL, et al. Phylogeny and origins of hantaviruses harbored by bats, insectivores, and rodents. PLoS Pathog. 2013; 9(2): e1003159. doi: 10.1371/journal.ppat.1003159 23408889 PMC3567184

[29] Weisburg WG, Barns SM, Pelletier DA, Lane DJ. 16S ribosomal DNA amplification for phylogenetic study. J Bacteriol. 1991; 173(2): 697–703. doi: 10.1128/jb.173.2.697-703.1991 1987160 PMC207061

[30] Wen B, Jian R, Zhang Y, Chen R. Simultaneous detection of *Anaplasma marginale* and a new *Ehrlichia* species closely related to *Ehrlichia chaffeensis* by sequence analyses of 16S ribosomal DNA in *Boophilus microplus* ticks from Tibet. J Clin Microbiol. 2002; 40(9): 3286–3290. doi: 10.1128/JCM.40.9.3286–3290.2002 12202567 PMC130830

[31] Guo WP, Tie WF, Meng S, Li D, Wang JL, Du LY, et al. Extensive genetic diversity of *Anaplasma bovis* in ruminants in Xi’an, China. Ticks Tick Borne Dis. 2020; 11(5): 101477. doi: 10.1016/j.ttbdis.2020.101477 32723632

[32] Ybañez AP, Matsumoto K, Kishimoto T, Inokuma H. Molecular analyses of a potentially novel *Anaplasma* species closely related to *Anaplasma phagocytophilum* detected in sika deer (*Cervus nippon yesoensis*) in Japan. Vet Microbiol. 2012; 157(1–2): 232–236. doi: 10.1016/j.vetmic.2011.12.001 22204789

[33] Rar VA, Livanova NN, Panov VV, Doroschenko EK, Pukhovskaya NM, Vysochina NP, et al. Genetic diversity of *Anaplasma* and *Ehrlichia* in the Asian part of Russia. Ticks Tick Borne Dis. 2010; 1(1): 57–65. doi: 10.1016/j.ttbdis.2010.01.002 21771512

[34] Sumner JW, Nicholson WL, Massung RF. PCR amplification and comparison of nucleotide sequences from the *groESL* heat shock operon of *Ehrlichia* species. J Clin Microbiol. 1997; 35(8): 2087–2092. doi: 10.1128/jcm.35.8.2087–2092.1997 9230387 PMC229908

[35] Hall TA. BioEdit: a user-friendly biological sequence alignment editor and analysis program for Windows 95/98/NT. Nucl Acids Symp Ser. 1999; 41: 95–98.

[36] Burland TG. DNASTAR’s Lasergene sequence analysis software. Methods Mol Biol. 2000; 132: 71–91. doi: 10.1385/1-59259-192-2:71 10547832

[37] Guindon S, Dufayard JF, Lefort V, Anisimova M, Hordijk W, Gascuel O. New algorithms and methods to estimate maximum-likelihood phylogenies: assessing the performance of PhyML 3.0. Syst Biol. 2010; 59(3): 307–321. doi: 10.1093/sysbio/syq010 20525638

[38] Kumar S, Stecher G, Tamura K. MEGA7: Molecular Evolutionary Genetics Analysis version 7.0 for bigger datasets. Mol Biol Evol. 2016; 33(7): 1870–1874. doi: 10.1093/molbev/msw054 27004904 PMC8210823

[39] Selmi R, Belkahia H, Dhibi M, Abdelaali H, Lahmar S, Ben Said M, et al. Zoonotic vector-borne bacteria in wild rodents and associated ectoparasites from Tunisia. Infect Genet Evol. 2021; 95: 105039. doi: 10.1016/j.meegid.2021.105039 34438095

[40] Sun J, Liu H, Yao XY, Zhang YQ, Lv ZH, Shao JW. Circulation of four species of Anaplasmataceae bacteria in ticks in Harbin, northeastern China. Ticks Tick Borne Dis. 2023; 14(3): 102136. doi: 10.1016/j.ttbdis.2023.102136 36736131

[41] Gofton AW, Waudby HP, Petit S, Greay TL, Ryan UM, Irwin PJ. Detection and phylogenetic characterisation of novel *Anaplasma* and *Ehrlichia* species in *Amblyomma triguttatum* subsp. from four allopatric populations in Australia. Ticks Tick Borne Dis. 2017; 8(5): 749–756. doi: 10.1016/j.ttbdis.2017.05.009 28601472

[42] Kosoy M, Bai Y, Sheff K, Morway C, Baggett H, Maloney SA, et al. Identification of *Bartonella* infections in febrile human patients from Thailand and their potential animal reservoirs. Am J Trop Med Hyg. 2010; 82(6): 1140–1145. doi: 10.4269/ajtmh.2010.09–0778 20519614 PMC2877425

[43] La Scola B, Zeaiter Z, Khamis A, Raoult D. Gene-sequence-based criteria for species definition in bacteriology: the *Bartonella* paradigm. Trends Microbiol. 2003; 11(7): 318–321. doi: 10.1016/s0966-842x(03)00143-4 12875815

[44] Chen M, Liu Q, Xue J, Chen S, Huang D, Yu Y, et al. Spreading of Human Babesiosis in China: Current Epidemiological Status and Future Challenges. China CDC Wkly. 2020; 2(33): 634–637. doi: 10.46234/ccdcw2020.176 34594726 PMC8392958

[45] Jia N, Zheng YC, Jiang JF, Jiang RR, Jiang BG, Wei R, et al. Human Babesiosis Caused by a *Babesia crassa*-Like Pathogen: A Case Series. Clin Infect Dis. 2018; 67(7): 1110–1119. doi: 10.1093/cid/ciy212 29538646

[46] Man SQ, Qiao K, Cui J, Feng M, Fu YF, Cheng XJ. A case of human infection with a novel *Babesia* species in China. Infect Dis Poverty. 2016; 5: 28. doi: 10.1186/s40249-016-0121-1 27025290 PMC4812642

[47] Saito-Ito A, Takada N, Ishiguro F, Fujita H, Yano Y, Ma XH, et al. Detection of Kobe-type *Babesia microti* associated with Japanese human babesiosis in field rodents in central Taiwan and southeastern mainland China. Parasitology. 2008; 135(6): 691–699. doi: 10.1017/S0031182008004356 18413002

[48] Wei F, Song M, Liu H, Wang B, Wang S, Wang Z, et al. Molecular Detection and Characterization of Zoonotic and Veterinary Pathogens in Ticks from Northeastern China. Front. Microbiol. 2016; 7: 1913. doi: 10.3389/fmicb.2016.01913 27965644 PMC5126052

[49] Zhao XG, Li H, Sun Y, Zhang YY, Jiang JF, Liu W, et al. Dual infection with *Anaplasma phagocytophilum* and *Babesia microti* in a *Rattus norvegicus*, China. Ticks Tick Borne Dis. 2013; 4(5): 399–402. doi: 10.1016/j.ttbdis.2013.04.002 23838022

[50] Zeng Z, Zhou S, Xu G, Liu W, Han T, Liu J, et al. Prevalence and phylogenetic analysis of *Babesia* parasites in reservoir host species in Fujian province, Southeast China. Zoonoses Public Health. 2022; 69(8): 915–924. doi: 10.1111/zph.12988 35819239

[51] Lu Y, Peng H, Zhu H, Li J, Xue S. Investigation of two blood parasitic protozoa infection in farmed *Macaca fascicularis* in Guangxi Zhuang Autonomous Region. Zhongguo Xue Xi Chong Bing Fang Zhi Za Zhi. 2016; 28(2): 141–145 (in Chinese). doi: 10.16250/j.32.1374.2015241 29469289

[52] Qiao Y, Peng H, Zhu H, Yan J. Nest-PCR identification of one human infected of *Babesia microti* in Guangxi and investigation on his colleagues. International Journal of Medical Parasitic Diseases. 2015; 42(3): 152–155 (in Chinese). doi: 10.3760/cma.j.issn.1673-4122.2015.03.007

[53] Wang H, Xu J, Peng H, Li M, Chen Y, Liu T, et al. Investigation of ticks and pathogen infection at a police dog breeding and training base in Nanning. International Journal of Medical Parasitic Diseases. 2015; 42(2): 94–98 (in Chinese). doi: 10.3760/cma.j.issn.1673-4122.2015.02.008

[54] Shuĭkina EE, Beĭer TV, Sergiev VP, Iastrebova RI. Detection of hemogregarin of the genus *Hepatozoon* in patients in Russia. Med Parazitol (Mosk). 2004; 28(4): 3–6 (in Russian). 15689126

[55] Demoner LC, Magro NM, da Silva MRL, de Paula Antunes JMA, Calabuig CIP, O’Dwyer LH. *Hepatozoon* spp. infections in wild rodents in an area of endemic canine hepatozoonosis in southeastern Brazil. Ticks Tick Borne Dis. 2016; 7(5): 859–864. doi: 10.1016/j.ttbdis.2016.04.002 27091081

[56] Johnson EM, Allen KE, Breshears MA, Panciera RJ, Little SE, Ewing SA. Experimental transmission of *Hepatozoon americanum* to rodents. Vet Parasitol. 2008; 151(2–4): 164–169. 10.1016/j.vetpar.2007.10.017. 18055118

[57] Han H, Wu Y, Dong H, Zhu S, Li L, Zhao Q, et al. First report of *Hepatozoon* (Apicomplexa: Adeleorina) from king ratsnakes (*Elaphe carinata*) in Shanghai, with description of a new species. Acta Parasitol. 2015; 60(2): 266–274. doi: 10.1515/ap-2015-0038 26203995

[58] Xiao X, Qi R, Han HJ, Liu JW, Qin XR, Fang LZ, et al. Molecular identification and phylogenetic analysis of *Cryptosporidium*, *Hepatozoon* and *Spirometra* in snakes from central China. Int J Parasitol Parasites Wildl. 2019; 10: 274–280. doi: 10.1016/j.ijppaw.2019.10.001 31700790 PMC6829678

[59] Guo WP, Xie GC, Xue ZQ, Yu JJ, Jian R, Du LY, et al. Molecular detection of *Hepatozoon canis* in dogs and ticks in Shaanxi province, China. Comp Immunol Microbiol Infect Dis. 2020; 72: 101514. doi: 10.1016/j.cimid.2020.101514 32634650

