# Supplementary material for: Great genetic diversity of vector-borne bacteria and protozoan in wild rodents from Guangxi, China
Source: PLoS Negl Trop Dis. 2024 May 13;18(5):e0012159. doi: 10.1371/journal.pntd.0012159 (PMC11115304; doi:10.1371/journal.pntd.0012159)
Supplement: S1 Table — (DOCX) [file pntd.0012159.s001.docx]

**Table S1** GenBank accession numbers of sequences obtained in this study.

|  | **Gene** | **Genbank numbers** | **Bacterial strain** |
| --- | --- | --- | --- |
| 1 | 16S rRNA | OR104942 | *Anaplasma capra* isolate GXS74 |
| 2 | 16S rRNA | OR104943 | *Anaplasma bovis* isolate GXS70 |
| 3 | 16S rRNA | OR104944 | Uncultured *Ehrlichia* sp. clone GXS19 |
| 4 | 16S rRNA | OR104945 | *Anaplasma ovis* isolate GXS45 |
| 5 | 16S rRNA | OR104946 | *Anaplasma ovis* isolate GXS56 |
| 6 | 16S rRNA | OR104947 | *Anaplasma ovis* isolate GXS14 |
| 7 | 16S rRNA | OR104948 | *Anaplasma ovis* isolate GXS5 |
| 8 | 16S rRNA | OR104949 | *Anaplasma bovis* isolate GXS6 |
| 9 | 16S rRNA | OR104950 | *Candidatus* Ehrlichia zunyiensis isolate CCBF3 |
| 10 | 16S rRNA | OR104951 | *Candidatus* Ehrlichia zunyiensis isolate CCBF4 |
| 11 | 16S rRNA | OR104953 | Uncultured *Ehrlichia* sp. clone GXS35 |
| 12 | 16S rRNA | OR104954 | Uncultured *Ehrlichia* sp. clone GXS50 |
| 13 | *gltA* | OR117471 | *Candidatus* Ehrlichia zunyiensis isolate CCBF3 |
| 14 | *gltA* | OR117472 | *Anaplasma phagocytophilum* isolate GXS61 |
| 15 | *gltA* | OR117473 | *Anaplasma phagocytophilum* isolate GXS21 |
| 16 | *gltA* | OR117474 | *Anaplasma phagocytophilum* isolate GXS33 |
| 17 | *gltA* | OR117475 | *Anaplasma bovis* isolate GXS27 |
| 18 | *ftsZ* | OR117477 | *Bartonella coopersplainsensis* isolate CCBF5 |
| 19 | *ftsZ* | OR117478 | *Bartonella tribocorum* isolate CCBF3 |
| 20 | *ftsZ* | OR117479 | *Bartonella tribocorum* isolate CCBF4 |
| 21 | *ftsZ* | OR117480 | *Bartonella tribocorum* isolate GXS1 |
| 22 | *ftsZ* | OR117481 | *Bartonella tribocorum* isolate GXS7 |
| 23 | *ftsZ* | OR117482 | *Bartonella tribocorum* isolate GXS8 |
| 24 | *ftsZ* | OR117483 | *Bartonella tribocorum* isolate GXS18 |
| 25 | *ftsZ* | OR117484 | *Bartonella tribocorum* isolate GXS20 |
| 26 | *ftsZ* | OR117485 | *Bartonella tribocorum* isolate GXS45 |
| 27 | *ftsZ* | OR117486 | *Bartonella tribocorum* isolate NM27 |
| 28 | *ftsZ* | OR117487 | *Bartonella tribocorum* isolate NM35 |
| 29 | *ftsZ* | OR117488 | *Bartonella tribocorum* isolate SS8 |
| 30 | *ftsZ* | OR117489 | *Bartonella tribocorum* isolate SS35 |
| 31 | *ftsZ* | OR117490 | *Bartonella rattimassiliensis* isolate GXS62 |
| 32 | *ftsZ* | OR117491 | *Bartonella rattimassiliensis* isolate GXS88 |
| 33 | *ftsZ* | OR117492 | *Candidatus* Bartonella fengshanensis isolate GXS56 |
| 34 | *ftsZ* | OR117493 | *Candidatus* Bartonella fengshanensis isolate GXS63 |
| 35 | *ftsZ* | OR117494 | *Candidatus* Bartonella fengshanensis isolate GXS64 |
| 36 | *ftsZ* | OR117495 | *Bartonella silvatica* isolate NM23 |
| 37 | *ftsZ* | OR117496 | *Bartonella silvatica* isolate NM26 |
| 38 | *ftsZ* | OR117497 | *Bartonella silvatica* isolate SS14 |
| 39 | *ftsZ* | OR117498 | *Bartonella silvatica* isolate SS18 |
| 40 | *ftsZ* | OR117499 | *Bartonella silvatica* isolate SS20 |
| 41 | *ftsZ* | OR117500 | *Bartonella silvatica* isolate SS37 |
| 42 | *ftsZ* | OR117501 | *Candidatus* Bartonella shangsiensis isolate SS7 |
| 43 | *ftsZ* | OR117502 | *Candidatus* Bartonella shangsiensis isolate SS21 |
| 44 | *ftsZ* | OR117503 | *Candidatus* Bartonella shangsiensis isolate SS27 |
| 45 | *ftsZ* | OR117504 | *Candidatus* Bartonella shangsiensis isolate SS29 |
| 46 | *ftsZ* | OR117505 | *Candidatus* Bartonella shangsiensis isolate SS31 |
| 47 | *ftsZ* | OR117506 | *Candidatus* Bartonella shangsiensis isolate SS33 |
| 48 | *groEL* | OR117507 | *Candidatus* Neoehrlichia mikurensis isolate SS13 |
| 49 | *groEL* | OR117508 | *Candidatus* Neoehrlichia mikurensis isolate SS47 |
| 50 | *groEL* | OR117509 | *Candidatus* Neoehrlichia mikurensis isolate GXS3 |
| 51 | *groEL* | OR117510 | *Candidatus* Neoehrlichia mikurensis isolate GXS70 |
| 52 | *groEL* | OR117511 | *Candidatus* Neoehrlichia mikurensis isolate GXS66 |
| 53 | *groEL* | OR117512 | *Candidatus* Neoehrlichia mikurensis isolate GXS43 |
| 54 | *groEL* | OR117513 | *Candidatus* Neoehrlichia mikurensis isolate GXS22 |
| 55 | *groEL* | OR117514 | *Candidatus* Neoehrlichia mikurensis isolate GXS21 |
| 56 | *groEL* | OR117515 | *Candidatus* Neoehrlichia mikurensis isolate GXS33 |
| 57 | *groEL* | OR117516 | *Candidatus* Neoehrlichia mikurensis isolate GXS10 |
| 58 | *groEL* | OR117517 | *Candidatus* Neoehrlichia mikurensis isolate GXS13 |
| 59 | *groEL* | OR117518 | *Candidatus* Neoehrlichia mikurensis isolate GXS16 |
| 60 | *groEL* | OR117519 | *Candidatus* Neoehrlichia mikurensis isolate GXS24 |
| 61 | *groEL* | OR117520 | *Candidatus* Neoehrlichia mikurensis isolate GXS26 |
| 62 | *groEL* | OR117521 | *Candidatus* Neoehrlichia mikurensis isolate GXS30 |
| 63 | *groEL* | OR117522 | *Candidatus* Neoehrlichia mikurensis isolate GXS91 |
| 64 | *groEL* | OR117523 | *Candidatus* Neoehrlichia mikurensis isolate GXS81 |
| 65 | *groEL* | OR117524 | *Candidatus* Neoehrlichia mikurensis isolate GXS71 |
| 66 | *groEL* | OR117525 | *Candidatus* Neoehrlichia mikurensis isolate GXS64 |
| 67 | *groEL* | OR117526 | *Candidatus* Neoehrlichia mikurensis isolate GXS63 |
| 68 | *groEL* | OR117527 | *Candidatus* Neoehrlichia mikurensis isolate GXS61 |
| 69 | *groEL* | OR117528 | *Candidatus* Neoehrlichia mikurensis isolate GXS55 |
| 70 | *groEL* | OR117529 | *Candidatus* Neoehrlichia mikurensis isolate GXS50 |
| 71 | *groEL* | OR117530 | *Candidatus* Neoehrlichia mikurensis isolate GXS49 |
| 72 | *groEL* | OR117531 | *Candidatus* Neoehrlichia mikurensis isolate GXS41 |
| 73 | *groEL* | OR117532 | *Candidatus* Neoehrlichia mikurensis isolate GXS35 |
| 74 | *groEL* | OR117533 | *Candidatus* Neoehrlichia mikurensis isolate GXS46 |
| 75 | *groEL* | OR117597 | *Anaplasma bovis* isolate SS7 |
| 76 | *groEL* | OR117598 | *Candidatus* Ehrlichia zunyiensis isolate CCBF3 |
| 77 | *groEL* | OR117599 | *Candidatus* Ehrlichia zunyiensis isolate CCBF4 |
| 78 | *groEL* | OR117600 | *Candidatus* Ehrlichia hainanensis isolate CCBF6 |
| 79 | *groEL* | OR117601 | Uncultured *Ehrlichia* sp. isolate GXS15 |
| 80 | *gltA* | OR117602 | *Bartonella coopersplainsensis* isolate CCBF5 |
| 81 | *gltA* | OR117606 | *Bartonella tribocorum* isolate GXS7 |
| 82 | *gltA* | OR117607 | *Bartonella tribocorum* isolate GXS8 |
| 83 | *gltA* | OR117610 | *Bartonella tribocorum* isolate GXS45 |
| 84 | *gltA* | OR117612 | *Bartonella tribocorum* isolate SS8 |
| 85 | *gltA* | OR117613 | *Bartonella tribocorum* isolate SS35 |
| 86 | *gltA* | OR117614 | *Bartonella silvatica* isolate SS14 |
| 87 | *gltA* | OR117615 | *Bartonella silvatica* isolate SS20 |
| 88 | *gltA* | OR117618 | *Candidatus* Bartonella shangsiensis isolate SS27 |
| 89 | *gltA* | OR117619 | *Candidatus* Bartonella shangsiensis isolate SS29 |
| 90 | *gltA* | OR117621 | *Candidatus* Bartonella fengshanensis isolate GXS63 |
| 91 | *gltA* | OR117622 | *Candidatus* Bartonella fengshanensis isolate GXS64 |
| 92 | 18S rRNA | OR117717 | *Babesia microti* isolate GXS6 |
| 93 | 18S r RNA | OR117718 | *Babesia microti* isolate GXS37 |
| 94 | 18S r RNA | OR117719 | *Babesia microti* isolate GXS51 |
| 95 | 18S r RNA | OR117720 | *Babesia microti* isolate GXS52 |
| 96 | 18S r RNA | OR117721 | *Babesia microti* isolate GXS54 |
| 97 | 18S r RNA | OR117722 | *Hepatozoon* sp. isolate GXS14 |
| 98 | 18S r RNA | OR117723 | *Hepatozoon* sp. isolate GXS28 |
| 99 | 18S r RNA | OR117724 | *Hepatozoon* sp. isolate GXS29 |
| 100 | 18S r RNA | OR117725 | *Hepatozoon* sp. isolate GXS31 |
| 101 | 18S r RNA | OR117726 | *Hepatozoon* sp. isolate GXS38 |
| 102 | 18S r RNA | OR117727 | *Hepatozoon* sp. isolate GXS39 |
| 103 | 18S r RNA | OR117728 | *Hepatozoon* sp. isolate GXS48 |
| 104 | 18S r RNA | OR117729 | *Hepatozoon* sp. isolate GXS53 |
| 105 | 18S r RNA | OR117730 | *Hepatozoon* sp. isolate GXS58 |
| 106 | 18S r RNA | OR117731 | *Hepatozoon* sp. isolate GXS60 |
