# Supplementary material for: Great genetic diversity of vector-borne bacteria and protozoan in wild rodents from Guangxi, China
Source: PLoS Negl Trop Dis. 2024 May 13;18(5):e0012159. doi: 10.1371/journal.pntd.0012159 (PMC11115304; doi:10.1371/journal.pntd.0012159)
Supplement: S2 Table — (DOCX) [file pntd.0012159.s002.docx]

**Table S2** Pathogens detected in rodents from Guangxi, China

|  | Code | Rodent species | Location | Pathogens |
| --- | --- | --- | --- | --- |
| 1 | GXS1 | *Rat. andamanensis* | Fengshan | *Bartonella tribocorum* |
| 2 | GXS2 | *Rat. losea* | Fengshan | - |
| 3 | GXS3 | *Rat. andamanensis* | Fengshan | *Candidatus* Neoehrlichia mikurensis |
| 4 | GXS4 | *Rat. andamanensis* | Fengshan | - |
| 5 | GXS5 | *Rat. andamanensis* | Fengshan | *Anaplasma ovis* |
| 6 | GXS6 | *Rat. andamanensis* | Fengshan | *Anaplasma bovis*, *Babesia microti* |
| 7 | GXS7 | *Rat. losea* | Fengshan | *Bartonella tribocorum* |
| 8 | GXS8 | *Rat. losea* | Fengshan | *Bartonella tribocorum* |
| 9 | GXS9 | *Rat. andamanensis* | Fengshan | - |
| 10 | GXS10 | *Rat. andamanensis* | Fengshan | *Candidatus* Neoehrlichia mikurensis |
| 11 | GXS11 | *Rat. andamanensis* | Fengshan | - |
| 12 | GXS12 | *Rat. andamanensis* | Fengshan | - |
| 13 | GXS13 | *Rat. andamanensis* | Fengshan | *Candidatus* Neoehrlichia mikurensis |
| 14 | GXS14 | *Rat. losea* | Fengshan | *Anaplasma ovis*, *Hepatozoon* sp. |
| 15 | GXS15 | *Rat. losea* | Fengshan | Uncultured *Ehrlichia* sp. |
| 16 | GXS16 | *Rat. andamanensis* | Fengshan | *Candidatus* Neoehrlichia mikurensis |
| 17 | GXS17 | *Rat. losea* | Fengshan | - |
| 18 | GXS18 | *Rat. losea* | Fengshan | *Bartonella tribocorum* |
| 19 | GXS19 | *Rat. andamanensis* | Fengshan | Uncultured *Ehrlichia* sp. |
| 20 | GXS20 | *Rat. losea* | Fengshan | *Bartonella tribocorum* |
| 21 | GXS21 | *Rat. andamanensis* | Fengshan | *Anaplasma phagocytophilum*, *Candidatus* Neoehrlichia mikurensis |
| 22 | GXS22 | *Mus caroli* | Fengshan | *Candidatus* Neoehrlichia mikurensis |
| 23 | GXS23 | *Rat. andamanensis* | Fengshan | - |
| 24 | GXS24 | *Rat. andamanensis* | Fengshan | *Candidatus* Neoehrlichia mikurensis |
| 25 | GXS25 | *Rat. losea* | Fengshan | - |
| 26 | GXS26 | *Rat. andamanensis* | Fengshan | *Candidatus* Neoehrlichia mikurensis |
| 27 | GXS27 | *Rat. andamanensis* | Fengshan | *Anaplasma bovis* |
| 28 | GXS28 | *Rat. losea* | Fengshan | *Hepatozoon* sp. |
| 29 | GXS29 | *Rat. losea* | Fengshan | *Hepatozoon* sp. |
| 30 | GXS30 | *Rat. losea* | Fengshan | *Candidatus* Neoehrlichia mikurensis |
| 31 | GXS31 | *Rat. losea* | Fengshan | *Hepatozoon* sp. |
| 32 | GXS32 | *Rat. andamanensis* | Fengshan | - |
| 33 | GXS33 | *Rat. andamanensis* | Fengshan | *Anaplasma phagocytophilum*, *Candidatus* Neoehrlichia mikurensis |
| 34 | GXS34 | *Rat. andamanensis* | Fengshan | - |
| 35 | GXS35 | *Rat. andamanensis* | Fengshan | Uncultured *Ehrlichia* sp., *Candidatus* Neoehrlichia mikurensis |
| 36 | GXS36 | *Rat. losea* | Fengshan | - |
| 37 | GXS37 | *Rat. andamanensis* | Fengshan | *Babesia microti* |
| 38 | GXS38 | *Rat. losea* | Fengshan | *Hepatozoon* sp. |
| 39 | GXS39 | *Rat. losea* | Fengshan | *Hepatozoon* sp. |
| 40 | GXS40 | *Rat. andamanensis* | Fengshan | - |
| 41 | GXS41 | *Rat. andamanensis* | Fengshan | *Candidatus* Neoehrlichia mikurensis |
| 42 | GXS42 | *Rat. andamanensis* | Fengshan | - |
| 43 | GXS43 | *Rat. andamanensis* | Fengshan | *Candidatus* Neoehrlichia mikurensis |
| 44 | GXS44 | *Rat. andamanensis* | Fengshan | - |
| 45 | GXS45 | *Rat. andamanensis* | Fengshan | *Anaplasma ovis*, *Bartonella tribocorum* |
| 46 | GXS46 | *Rat. andamanensis* | Fengshan | *Candidatus* Neoehrlichia mikurensis |
| 47 | GXS47 | *Mus caroli* | Fengshan | - |
| 48 | GXS48 | *Rat. losea* | Fengshan | *Hepatozoon* sp. |
| 49 | GXS49 | *Rat. andamanensis* | Fengshan | *Candidatus* Neoehrlichia mikurensis |
| 50 | GXS50 | *Rat. andamanensis* | Fengshan | Uncultured *Ehrlichia* sp., *Candidatus* Neoehrlichia mikurensis |
| 51 | GXS51 | *Mus pahari* | Fengshan | *Babesia microti* |
| 52 | GXS52 | *Rat. andamanensis* | Fengshan | *Babesia microti* |
| 53 | GXS53 | *Rat. losea* | Fengshan | *Hepatozoon* sp. |
| 54 | GXS54 | *Mus pahari* | Fengshan | *Babesia microti* |
| 55 | GXS55 | *Rat. andamanensis* | Fengshan | *Candidatus* Neoehrlichia mikurensis |
| 56 | GXS56 | *Rat. losea* | Fengshan | *Anaplasma ovis*, *Candidatus* Bartonella fengshanensis |
| 57 | GXS57 | *Rat. losea* | Fengshan | - |
| 58 | GXS58 | *Rat. andamanensis* | Fengshan | *Hepatozoon* sp. |
| 59 | GXS59 | *Rat. andamanensis* | Fengshan | - |
| 60 | GXS60 | *Rat. losea* | Fengshan | *Hepatozoon* sp. |
| 61 | GXS61 | *Rat. andamanensis* | Fengshan | *Anaplasma phagocytophilum*, *Candidatus* Neoehrlichia mikurensis |
| 62 | GXS62 | *Rat. losea* | Fengshan | *Bartonella rattimassiliensis* |
| 63 | GXS63 | *Rat. losea* | Fengshan | *Candidatus* Bartonella fengshanensis, *Candidatus* Neoehrlichia mikurensis |
| 64 | GXS64 | *Rat. losea* | Fengshan | *Candidatus* Bartonella fengshanensis, *Candidatus* Neoehrlichia mikurensis |
| 65 | GXS65 | *Mus pahari* | Fengshan | - |
| 66 | GXS66 | *Rat. andamanensis* | Fengshan | *Candidatus* Neoehrlichia mikurensis |
| 67 | GXS67 | *Mus pahari* | Fengshan | - |
| 68 | GXS68 | *Mus pahari* | Fengshan | - |
| 69 | GXS69 | *Rat. losea* | Fengshan | - |
| 70 | GXS70 | *Rat. andamanensis* | Fengshan | *Anaplasma bovis*, *Candidatus* Neoehrlichia mikurensis |
| 71 | GXS71 | *Rat. andamanensis* | Fengshan | *Candidatus* Neoehrlichia mikurensis |
| 72 | GXS72 | *Rat. andamanensis* | Fengshan | - |
| 73 | GXS73 | *Rat. andamanensis* | Fengshan | - |
| 74 | GXS74 | *Mus pahari* | Fengshan | *Anaplasma capra* |
| 75 | GXS75 | *Mus pahari* | Fengshan | - |
| 76 | GXS76 | *Mus caroli* | Fengshan | - |
| 77 | GXS77 | *Rat. losea* | Fengshan | - |
| 78 | GXS78 | *Mus pahari* | Fengshan | - |
| 79 | GXS79 | *Rat. andamanensis* | Fengshan | - |
| 80 | GXS80 | *Mus pahari* | Fengshan | - |
| 81 | GXS81 | *Rat. andamanensis* | Fengshan | *Candidatus* Neoehrlichia mikurensis |
| 82 | GXS82 | *Rat. andamanensis* | Fengshan | - |
| 83 | GXS83 | *Rat. andamanensis* | Fengshan | - |
| 84 | GXS84 | *Rat. andamanensis* | Fengshan | - |
| 85 | GXS85 | *Mus pahari* | Fengshan | - |
| 86 | GXS86 | *Rat. andamanensis* | Fengshan | - |
| 87 | GXS87 | *Rat. andamanensis* | Fengshan | - |
| 88 | GXS88 | *Rat. losea* | Fengshan | *Bartonella rattimassiliensis* |
| 89 | GXS91 | *Rat. andamanensis* | Fengshan | *Candidatus* Neoehrlichia mikurensis |
| 90 | BF1 | *Ban. indica* | Fengshan | - |
| 91 | BF2 | *Ber. bowersi* | Fengshan | - |
| 92 | BF3 | *Ber. bowersi* | Fengshan | - |
| 93 | BF4 | *Ban. indica* | Fengshan | - |
| 94 | BF5 | *Ban. indica* | Fengshan | - |
| 95 | BF6 | *Ban. indica* | Fengshan | - |
| 96 | BF7 | *Ber. bowersi* | Fengshan | - |
| 97 | CCBF1 | *Leo. edwardsi* | Fengshan | - |
| 98 | CCBF2 | *Leo. edwardsi* | Fengshan | - |
| 99 | CCBF3 | *Leo. edwardsi* | Fengshan | *Candidatus* Ehrlichia zunyiensis, *Bartonella tribocorum* |
| 100 | CCBF4 | *Leo. edwardsi* | Fengshan | *Candidatus* Ehrlichia zunyiensis, *Bartonella tribocorum* |
| 101 | CCBF5 | *Leo. edwardsi* | Fengshan | *Bartonella coopersplainsensis* |
| 102 | CCBF6 | *Leo. edwardsi* | Fengshan | *Candidatus* Ehrlichia hainanensis |
| 103 | CCBF7 | *Leo. edwardsi* | Fengshan | - |
| 104 | CCBF8 | *Leo. edwardsi* | Fengshan | - |
| 105 | CCBF9 | *Leo. edwardsi* | Fengshan | - |
| 106 | NM1 | *Ban. indica* | Ningming | - |
| 107 | NM2 | *Ban. indica* | Ningming | *-* |
| 108 | NM3 | *Ban. indica* | Ningming | *-* |
| 109 | NM4 | *Ban. indica* | Ningming | *-* |
| 110 | NM5 | *Ban. indica* | Ningming | *-* |
| 111 | NM6 | *Ban. indica* | Ningming | *-* |
| 112 | NM7 | *Ban. indica* | Ningming | *-* |
| 113 | NM8 | *Ban. indica* | Ningming | *-* |
| 114 | NM9 | *Ban. indica* | Ningming | *-* |
| 115 | NM10 | *Ban. indica* | Ningming | *-* |
| 116 | NM11 | *Ban. indica* | Ningming | *-* |
| 117 | NM12 | *Ban. indica* | Ningming | *-* |
| 118 | NM13 | *Ban. indica* | Ningming | *-* |
| 119 | NM14 | *Ban. indica* | Ningming | *-* |
| 120 | NM15 | *Ban. indica* | Ningming | *-* |
| 121 | NM16 | *Ban. indica* | Ningming | *-* |
| 122 | NM17 | *Ban. indica* | Ningming | *-* |
| 123 | NM18 | *Ban. indica* | Ningming | *-* |
| 124 | NM19 | *Ban. indica* | Ningming | *-* |
| 125 | NM20 | *Ban. indica* | Ningming | *-* |
| 126 | NM21 | *Ban. indica* | Ningming | *-* |
| 127 | NM22 | *Ban. indica* | Ningming | *-* |
| 128 | NM23 | *Ban. indica* | Ningming | *Bartonella silvatica* |
| 129 | NM24 | *Ban. indica* | Ningming | *-* |
| 130 | NM25 | *Ban. indica* | Ningming | *-* |
| 131 | NM26 | *Ban. indica* | Ningming | *Bartonella silvatica* |
| 132 | NM27 | *Ban. indica* | Ningming | *Bartonella tribocorum* |
| 133 | NM28 | *Ban. indica* | Ningming | *-* |
| 134 | NM29 | *Ban. indica* | Ningming | *-* |
| 135 | NM30 | *Ban. indica* | Ningming | *-* |
| 136 | NM31 | *Ban. indica* | Ningming | *-* |
| 137 | NM32 | *Ban. indica* | Ningming | *-* |
| 138 | NM33 | *Ban. indica* | Ningming | *-* |
| 139 | NM34 | *Ban. indica* | Ningming | *-* |
| 140 | NM35 | *Ban. indica* | Ningming | *Bartonella tribocorum* |
| 141 | NM36 | *Ban. indica* | Ningming | *-* |
| 142 | NM37 | *Ban. indica* | Ningming | *-* |
| 143 | NM38 | *Ban. indica* | Ningming | *-* |
| 144 | SS1 | *Ban. indica* | Shangsi | *-* |
| 145 | SS2 | *Ban. indica* | Shangsi | *-* |
| 146 | SS3 | *Ban. indica* | Shangsi | *-* |
| 147 | SS4 | *Ban. indica* | Shangsi | *-* |
| 148 | SS5 | *Ban. indica* | Shangsi | *-* |
| 149 | SS6 | *Ban. indica* | Shangsi | *-* |
| 150 | SS7 | *Ban. indica* | Shangsi | *Candidatus* Bartonella shangsiensis, *Anaplasma bovis* |
| 151 | SS8 | *Ban. indica* | Shangsi | *Bartonella tribocorum* |
| 152 | SS9 | *Ban. indica* | Shangsi | *-* |
| 153 | SS10 | *Ban. indica* | Shangsi | *-* |
| 154 | SS11 | *Ban. indica* | Shangsi | *-* |
| 155 | SS12 | *Ban. indica* | Shangsi | *-* |
| 156 | SS13 | *Ban. indica* | Shangsi | *Candidatus* Neoehrlichia mikurensis |
| 157 | SS14 | *Ban. indica* | Shangsi | *Bartonella silvatica* |
| 158 | SS15 | *Ban. indica* | Shangsi | *-* |
| 159 | SS16 | *Ban. indica* | Shangsi | *-* |
| 160 | SS17 | *Ban. indica* | Shangsi | *-* |
| 161 | SS18 | *Ban. indica* | Shangsi | *Bartonella silvatica* |
| 162 | SS19 | *Ban. indica* | Shangsi | *-* |
| 163 | SS20 | *Ban. indica* | Shangsi | *Bartonella silvatica* |
| 164 | SS21 | *Ban. indica* | Shangsi | *Candidatus* Bartonella shangsiensis |
| 165 | SS22 | *Ban. indica* | Shangsi | *-* |
| 166 | SS23 | *Ban. indica* | Shangsi | *-* |
| 167 | SS24 | *Ban. indica* | Shangsi | *-* |
| 168 | SS25 | *Ban. indica* | Shangsi | *-* |
| 169 | SS26 | *Ban. indica* | Shangsi | *-* |
| 170 | SS27 | *Ban. indica* | Shangsi | *Candidatus* Bartonella shangsiensis |
| 171 | SS28 | *Ban. indica* | Shangsi | *-* |
| 172 | SS29 | *Ban. indica* | Shangsi | *Candidatus* Bartonella shangsiensis |
| 173 | SS30 | *Ban. indica* | Shangsi | *-* |
| 174 | SS31 | *Ban. indica* | Shangsi | *Candidatus* Bartonella shangsiensis |
| 175 | SS32 | *Ban. indica* | Shangsi | *-* |
| 176 | SS33 | *Ban. indica* | Shangsi | *Candidatus* Bartonella shangsiensis |
| 177 | SS34 | *Ban. indica* | Shangsi | *-* |
| 178 | SS35 | *Ban. indica* | Shangsi | *Bartonella tribocorum* |
| 179 | SS36 | *Ban. indica* | Shangsi | *-* |
| 180 | SS37 | *Ban. indica* | Shangsi | *Bartonella silvatica* |
| 181 | SS38 | *Ban. indica* | Shangsi | *-* |
| 182 | SS39 | *Ban. indica* | Shangsi | *-* |
| 183 | SS40 | *Ban. indica* | Shangsi | *-* |
| 184 | SS41 | *Ban. indica* | Shangsi | *-* |
| 185 | SS42 | *Ban. indica* | Shangsi | *-* |
| 186 | SS43 | *Ban. indica* | Shangsi | *-* |
| 187 | SS44 | *Ban. indica* | Shangsi | *-* |
| 188 | SS45 | *Ban. indica* | Shangsi | *-* |
| 189 | SS46 | *Ban. indica* | Shangsi | *-* |
| 190 | SS47 | *Ban. indica* | Shangsi | *Candidatus* Neoehrlichia mikurensis |
| 191 | SS48 | *Ban. indica* | Shangsi | *-* |
| 192 | SS49 | *Ban. indica* | Shangsi | *-* |
